# Subtype‐specific enhancer RNAs define transcriptional regulators and prognosis in breast cancers

**DOI:** 10.1002/1878-0261.70214

**Published:** 2026-03-02

**Authors:** Aamena Y. Patel, Peyman Zarrineh, Nathnael T. Tuffa, Jigar H. Sheth, Sumitra Mohan, Mudassar Iqbal, Sankari Nagarajan

**Affiliations:** ^1^ Division of Molecular and Cellular Function, School of Biological Sciences, Faculty of Biology, Medicine and Health The University of Manchester UK; ^2^ Division of Cancer Sciences, School of Medical Sciences, Faculty of Biology, Medicine and Health The University of Manchester UK; ^3^ Bioinformatics Core Facility, Faculty of Biology, Medicine and Health The University of Manchester UK; ^4^ Division of Informatics, Imaging and Data Sciences, School of Health Sciences, Faculty of Biology, Medicine and Health The University of Manchester UK

**Keywords:** breast cancer, enhancer RNA, information gain, machine learning, random forest

## Abstract

Gene expression is tightly controlled by DNA elements called enhancers by associating with lineage‐specific transcription factors. These enhancers transcribe noncoding RNAs (called enhancer RNAs or eRNAs). eRNA expression is an early indicator of transcription factor activity and is associated with treatment response and survival in cancer patients. By analysing ~ 300 000 eRNA loci profiled using RNA‐sequencing data sets from 975 breast cancer patients using machine learning approaches, we categorised eRNAs specific to breast cancer molecular subtypes and survival. We associated these eRNAs with subtype‐specific mRNAs to define proximal co‐expressed regulatory eRNAs (ProxCReAm), which are enriched in pathways characteristic of their respective subtypes. Interestingly, cistrome and transcription factor motif analyses on these eRNAs highlighted involvement of diverse nuclear receptors (GR/AHR for luminal and GR/RAR for basal) and FOX factors in luminal regions. Moreover, luminal eRNAs were associated with better outcomes and Her2 eRNAs with worse outcomes in patients. Overall, we demonstrate that machine learning approaches performed on RNA‐seq data sets can classify functionally relevant subtype‐specific and prognostic eRNAs, which can identify critical gene pathways and transcription factor networks in breast cancer.

## Graphical abstract

This study employed machine learning methodologies to perform the subtype‐specific classification of RNA‐seq data sets, which are mapped on enhancers from TCGA‐derived breast cancer patients. Their integration with gene expression (referred to as ProxCReAM eRNAs) and chromatin accessibility profiles has the potential to identify lineage‐specific and relevant transcriptional regulators and to associate with patient survival more efficiently.

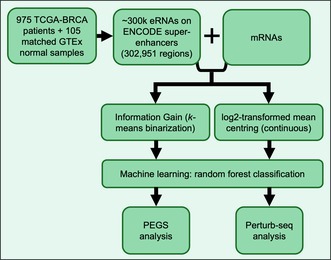

**Abbreviations**
AHR: aryl hydrocarbon receptor
AME: Analysis of Motif Enrichment
ATAC: assay for transposase‐accessible chromatin
CAGE: Cap Analysis of Gene Expression
CGP: chemical and genetic perturbations
CHD8: Chromodomain‐helicase DNA binding protein
ChIP: chromatin immunoprecipitation
CPM: counts per million
CREBBP: Cyclic AMP response element‐binding protein
ER: oestrogen receptor
eRNAs: enhancer RNAs
FN: false negative
FP: false positive
GR: glucocorticoid receptor
GRO‐seq: Global Run‐on sequencing
Her2: human epidermal growth factor receptor‐2
HIF1A: Hypoxia‐inducible factor 1 alpha
InfoGain: information gain
KAS‐seq: kethoxal‐assisted single‐stranded DNA sequencing
Logmc: log mean centring
MAD: median‐absolute‐deviation
PC: principal component
PCA: principal component analysis
PEGS: Peak set Enrichment in Gene Sets
PR: progesterone receptor
ProxCReAms: Proximally Co‐expressed Regulatory eRNAs, which are Associated with mRNAs
RNAPII: RNA polymerase II
Rpkm: reads per kilobase per million mapped reads
SMARCA4: SWI/SNF Related BAF Chromatin Remodelling Complex Subunit ATPase‐4
SPI1: Spi‐1 protooncogene
TADs: topologically associated domains
TCeA: TCGA eRNA atlas
TN: true negative
TP: true positive
UMAP: Uniform Manifold Approximation and Projection for Dimension Reduction

## 1 Introduction

Breast cancer is a highly heterogenous disease representing various subtypes with differences in the expression of classical hormonal and growth factor receptors, anatomical origin and gene alterations. Based on the expression of receptors‐ oestrogen receptor (ER), progesterone receptor (PR) and human epidermal growth factor receptor‐2 (Her2), pathologists classify breast tumours into different subtypes to enable targeted therapies. The molecular subtypes are hence classified as Luminal A (low‐proliferating ER+ PR+ Her2‐), Luminal B (highly proliferating ER+ PR+ Her2+/−), Her2 (Her2+ alone), basal (majorly triple‐negative ER‐ PR‐ Her2‐) and normal‐like [1, 2]. The prognosis of each subtype‐specific patient is distinct [3], emphasising the importance of these stratifications. LumA patients show a better prognosis than LumB, while Her2 and basal patients have the worst outcomes. Overall, this highlights the importance of stratification of breast cancer patients at the molecular level to obtain efficient therapeutic benefits and better survival. Furthermore, breast tumours originate from either ducts and/or milk lobules in the breast. Lobular cancers are the least studied breast cancers and have a worse prognosis than ductal cancers due to their aggressiveness and drug resistance to ER‐targeting therapies. Despite the major histological changes such as discohesive morphology of lobular cancers, identifying their molecular differences is cumbersome, hindering the development of specific therapeutic strategies.

Gene transcription is highly regulated by *cis*‐regulatory elements called enhancers. These are regions which can be located several 100–1000 s of kilobases away from gene promoters but can control gene expression via 3D chromosomal interactions. Enhancers are bound and driven by lineage‐specific transcription factors. Various studies in breast cancers showed expression of noncoding RNAs from highly active enhancers, termed as enhancer RNAs (eRNAs) [4, 5, 6]. eRNA expression precedes corresponding gene transcription, thus representing an upstream regulatory mechanism and is correlated with high enhancer activity [7, 8, 9]. Importantly, eRNA expression is highly correlated with the activity of transcription factors such as ERα [4, 5], FOXA1 [10], AR [11] and p53 [12]. Hence, enhancers which produce eRNAs can be evaluated to identify the transcription factors which are bound to and involved in driving the activity of those regions, in an unbiased manner [6]. However, identifying eRNAs in patient samples is difficult, as the majority of eRNAs are nonpolyadenylated and therefore are unstable. Hence, their detection requires high sample input and laborious techniques. As such, developing robust assays to identify eRNA expression in patient samples would be highly beneficial in developing patient‐specific molecular signatures and key transcription factor networks.

Existing pan‐cancer studies based on deeply sequenced and aggregated RNA‐seq data sets identified cancer type‐specific polyadenylated eRNAs, which are more prognostic than mRNAs in certain types of cancer [13, 14]. These eRNAs represent strong super‐enhancer activity and can determine immunotherapeutic response in a cell‐specific manner, resolving intra‐tumour heterogeneity. Altogether, these findings suggest that eRNAs identified from RNA‐seq data sets can provide sufficient power to be associated with patient survival. Despite the identification of 326 prognostic eRNAs in breast cancer from these studies, they are less prognostic than mRNAs. However, the heterogeneous nature of breast cancers has not been considered in their analyses, and this warrants further investigation following appropriate classification of patient datasets based on molecular subtypes.

In this study, we employed machine learning approaches on 302 951 eRNA loci identified from RNA‐seq datasets from 975 breast cancer patient samples from previous studies [13, 14]. We identified eRNAs and mRNAs which are specific to subtypes and survival using machine learning approaches and associated the subtype‐specific eRNAs with proximal co‐expressed subtype‐specific mRNAs (ProxCReAms, Proximally Co‐expressed Regulatory eRNAs, which are Associated with mRNAs), to define the key eRNAs in breast cancers. In addition to the major differences observed in molecular subtypes, our work identified that eRNAs are associated with key gene pathways and transcription factors specific to each subtype. Overall, our results highlight the utility of stratified approaches on eRNA expression for understanding the important pathways involved in cancer progression.

## 2 Materials and methods

### 2.1 Data sets

eRNA expression data sets from 1095 breast cancer patient samples were downloaded from TCGA eRNA atlas (TCeA) platform [14]. These were mapped on 302 951 enhancer loci, which were identified from H3K27ac chromatin immunoprecipitation (ChIP)‐sequencing datasets from Hnisz *et al*. [15]. The patient metadata was downloaded from TCGA website https://portal.gdc.cancer.gov/projects/TCGA‐BRCA. Out of the 1095 breast cancer samples, 120 samples expressing either extremely low or high levels of eRNA were filtered using outlier detection method with median‐absolute‐deviation (MAD) using scater package (v1.22.0) [16], with number of MADs away from median required (nmads) as 1.5. Data from remaining 975 breast cancer tumour samples were used for further classification approaches.

For the Cistrome‐based binding overlap analysis [17], data were downloaded from the section ‘TF ChIP‐seq signals on eRNA loci’ under ‘Integrated analysis’ from TCeA. ChIP‐seq and Global Run‐on (GRO‐seq) data sets were downloaded from published data sets for ER [18, 19], H3K27ac [20], RNA polymerase [21] and GRO‐seq from 40 min oestrogen‐treated MCF7 cells [4]. Assay for transposase‐accessible chromatin (ATAC)‐seq data sets were utilised from the TCGA breast cancer cohort [22]. Cap Analysis of Gene Expression (CAGE) data set from MCF7 cells was accessed from published data sets (GSM3318135) [23]. Perturb‐seq data sets of two breast cancer‐related cell lines were downloaded from Wang *et al*. [24].

### 2.2 Subtype‐specific eRNA detection, dimension reduction and classification

Binary values (expressed or nonexpressed eRNAs) were generated by applying *k*‐means binning with *k* = 2 on log2 reads per kilobase per million mapped reads (rpkm) eRNA expression values. fselector (v0.34) [25] package was used to calculate the information gain (InfoGain) values associated with the features (eRNAs) across the comparison sets (subtypes) and 0.05 cut‐off was used for the binary expression values. As Her2 survival‐specific eRNAs with 0.05 cut‐off were over 7000 loci, a stringent cut‐off (0.1) was used for survival‐specific classification (living vs deceased patients as good vs poor outcome respectively, based on the ‘Overall survival status’ of the patients). Average log2‐transformed mean‐centred rpkm values of eRNA (log mean centring or Logmc) over 1.0 cut‐off were selected as continuous values.

Principal component analysis (PCA) plots were visualised using the *prcomp* function from *stats* in r (v4.5.1) using principal components (PCs) 1 and 2. umap (v.0.2.10.0) [26] was used to visualise Uniform Manifold Approximation and Projection for Dimension Reduction (UMAP) plots with PC1 and PC2 for InfoGain and PC1‐4 for Logmc. randomforest (v4.7‐1.1) [27, 28] was used to perform classifications into subtypes or survival status. The data sets were divided into 70% training and 30% test sets.

The random forest classifications were statistically assessed using different sets of subtype‐ or survival‐specific eRNAs. These statistics described below use true positive (TP), true negative (TN), false positive (FP) and false negative (FN) metrics:

- Specificity = TN/(TN + FP).
- Sensitivity or Recall = TP/(TP + FN).
- Precision = TP/(TP + FP).
- Accuracy = (TP + TN)/(TP + FP + TN + FN).
- F‐measure = 2*(precision*Recall)/(precision + Recall).

### 2.3 Peak set enrichment in gene sets (PEGS) analysis

To identify the ProxCReAm eRNAs and the mRNA‐eRNA pairs, Peak set Enrichment in Gene Sets (PEGS) analysis was performed [29]. Mutual enrichment of subtype‐specific mRNAs (gene sets) with subtype‐specific eRNAs (peak sets) was computed across different genomic distances (150 bp–2 Mb) so that the appropriate genomic distance showing significant association could be selected. *P*‐values were calculated using hypergeometric test similar to the GREAT platform [30]. The distance (1 Mb) was chosen as the threshold distance, above which the *P*‐values substantially increased.

### 2.4 Pathway analysis

GSEA analysis was performed using *Molecular signature database* with chemical and genetic perturbations (CGP), C5 and C6 gene sets. enrichr analysis was performed using default settings for the Perturb‐seq overlapped enhancer‐associated genes [31].

### 2.5 KAS‐sequencing and analyses

Low input N3‐kethoxal‐assisted single‐stranded DNA sequencing (KAS‐seq) protocol was performed as described in Lyu *et al*. [32]. MCF7 cells (*n* = 2) were cultured with DMEM with 10% fetal bovine serum, penicillin, streptomycin and 2 mm glutamine in a 12‐well plate and labelled with 5 mm N3‐kethoxal for 10 min. After labelling, 10 000 labelled cells were collected for further reactions. For tagmentation reaction, 50 ng of biotinylated DNA and 7.5 μL of the enzyme from Illumina Tagment DNA TDE1 enzyme and buffer kit were used. The transposed and amplified DNA samples were sequenced using NovaSeq 6000 with 1% PhiX spike‐in, generating paired‐end reads. The reads were mapped on hg38 using bowtie2 [33] and macs2 (v2.2.7.1) broadpeaks option was used for peak calling [34]. deeptools (v3.5.1) [35] was used to construct bigwig files with rpkm settings (bamcoverage), average density plots (computematrix and plotprofile) and heatmaps (computematrix and plotheatmap). Distal KAS‐seq positive regions were calculated from all KAS‐seq peaks ±5 kb away from gene promoters and gene bodies after removing the blacklisted regions for hg38 (accession number ENCFF356LFX) [36] and sequencing artefacts.

### 2.6 Transcription factor enrichment and ATAC‐seq data analyses

To find the transcriptional or epigenetic factors which are significantly enriched near the eRNA loci, we overlapped the subtype‐specific eRNAs with the enhancer loci annotated with transcription factor binding from Cistrome‐based ChIP‐seq datasets [14, 17]. Total unique binding sites were calculated for each factor, irrespective of data from different cell lines from various cancers. *P*‐values were calculated using the phyper function from the *Hypergeometric distribution* in r stats (v4.4.2).

Lift over of ATAC‐seq datasets from hg38 to hg19 was performed using crossmap (v.2.0.0) and liftover (v1.0) [37, 38]. Peak calling was performed using macs2 v2.2.9.1 [39] bdgpeakcall function after converting the bigwig files to bedgraph using bigwigtobedgraph (version: kent source v489) [40]. The resulting bed files were merged according to their molecular subtypes using bedtools v2.31.1 merge function [41]. For motif enrichment, we initially performed the overlap analysis between ATAC‐seq peaks and eRNA peaks (with a 1‐kb flank on both sides). From the resulting accessible regions, we filtered out those annotated as exons, promoters and first introns. For motif enrichment analysis, *AME* (analysis of motif enrichment) v5.5.7 [42] was used. Bed files were created with ±1000 bp flanks of the ATAC‐seq peaks, which overlapped with the subtype‐specific eRNA summits as described above. These regions were compared to motifs in the *JASPAR 2022* Core vertebrates (nonredundant) database v2. For the background, the default shuffled input sequences were used. The adjusted *P*‐value and the ratio of true‐positive over false‐positive scores were summarised as average values per each motif family.

### 2.7 Survival analysis

Kaplan–Meier plots were performed using survival (v3.7‐0) [43, 44] and visualised with survminer (v0.4.9) [45] and ggplot2 (v3.5.1) [46] in r (v4.4.1). *P*‐values were calculated using the log‐rank test. Expression levels of subtype/survival‐specific eRNAs were categorised as high or low by separating the average of eRNA expression among patients as above or below 0, respectively. For the basal subtype, highly expressed eRNAs were considered, and thus, the median of the average eRNA expression was used to stratify patients into high‐ and low‐expression groups.

### 2.8 Visualisation

tidyverse (v2.0.0) [47] was utilised for data manipulation in r (v4.4.2). heatmaps were made using complexheatmap (v2.22.0) in r with heatmaps annotations [48, 49]. Genes with low counts across samples were filtered out prior to visualisation. HiC data sets were visualised using 3D Genome Browser 2.0 [50] using data sets from GSE116694 (HCC1954, Her2+ breast cancer cell line) [51] and GSE109229 (BT474, ER+ PR+ Her2+ breast cancer cell line) [52] with hg38 genome build. For the heatmaps with distal KAS‐seq peaks, the regions were sorted according to the intensity of the KAS‐seq signal in descending order. ggplot2 (v3.5.1) [46] was used to generate PCA plots and cowplot (v1.1.3) [53] was used to plot the grids with the dendrograms for the dot plots. Hierarchical clustering was performed using *hclust* function from r stats package (v4.4.2). The dendrograms were visualised using ggtree (v3.14.0) [54] and joined using patchwork (v1.3.0) [55]. Venn diagrams were created using eulerr (v7.0.2) [56, 57]. Annotation of genomic regions and the genomic distributions were profiled using chipseeker (v1.8.6) [58].

### 2.9 Perturb‐seq analysis

We overlapped the survival and subtype‐specific mRNAs and eRNA with the enhancers interrogated on the published Perturb‐seq data from breast cancer cell lines [24]. For this overlap, we used the subtype−/survival‐specific eRNA regions with 1 kb flanks, converted them to hg38 and then intersected them with Perturb‐seq evaluated regions. To identify the downstream altered genes of the selected eRNA and mRNA regions in the given perturb‐seq data, we downloaded the results of pySpade method developed by Wang *et al*. from the GEO database (accession number GSE224986). We used direct (local) and global gene target files, which contain the Perturb‐seq results for the MDA‐MB‐231 cell line, representing basal breast cancers, and the MDA‐MB‐361 cell line, representing luminal B breast cancers. The gene targets were matched within a 1 MB distance of the enhancers. In each data set, we subtracted the log‐transformed counts per million (CPM) values of perturbations from the log‐transformed CPM values of background for our regions of interest. Then, we applied 0.5 as a cutoff to detect perturbed genes per condition.

## 3 Results

### 3.1 Identification of subtype‐specific eRNAs

Previous work by Chen *et al*. [13] identified a weak association of eRNA expression with patient survival in breast cancers. Given the heterogeneity and different subtypes of breast cancer, we hypothesised that eRNAs can associate with patient survival depending on subtype. Hence, we attempted to define eRNAs based on molecular subtypes (basal, luminal A/B and Her2) and histological tissue type (ductal vs lobular) and relate these subtype‐specific eRNAs to survival. We reanalysed the datasets from Chen *et al*. [14] in which they mapped the eRNA signals in rpkm from 975 breast tumour samples on the high‐resolution super‐enhancer‐based eRNA loci (*n* = ~ 300 K loci). These regions were derived from ENCODE H3K27ac occupancy data sets from 86 human cell lines and tissue samples, representing active enhancers [15]. Hence, our analyses on ~ 300 K eRNA loci can provide unbiased information on subtype specificity and gene‐TF regulatory networks.

**Fig. 1:**
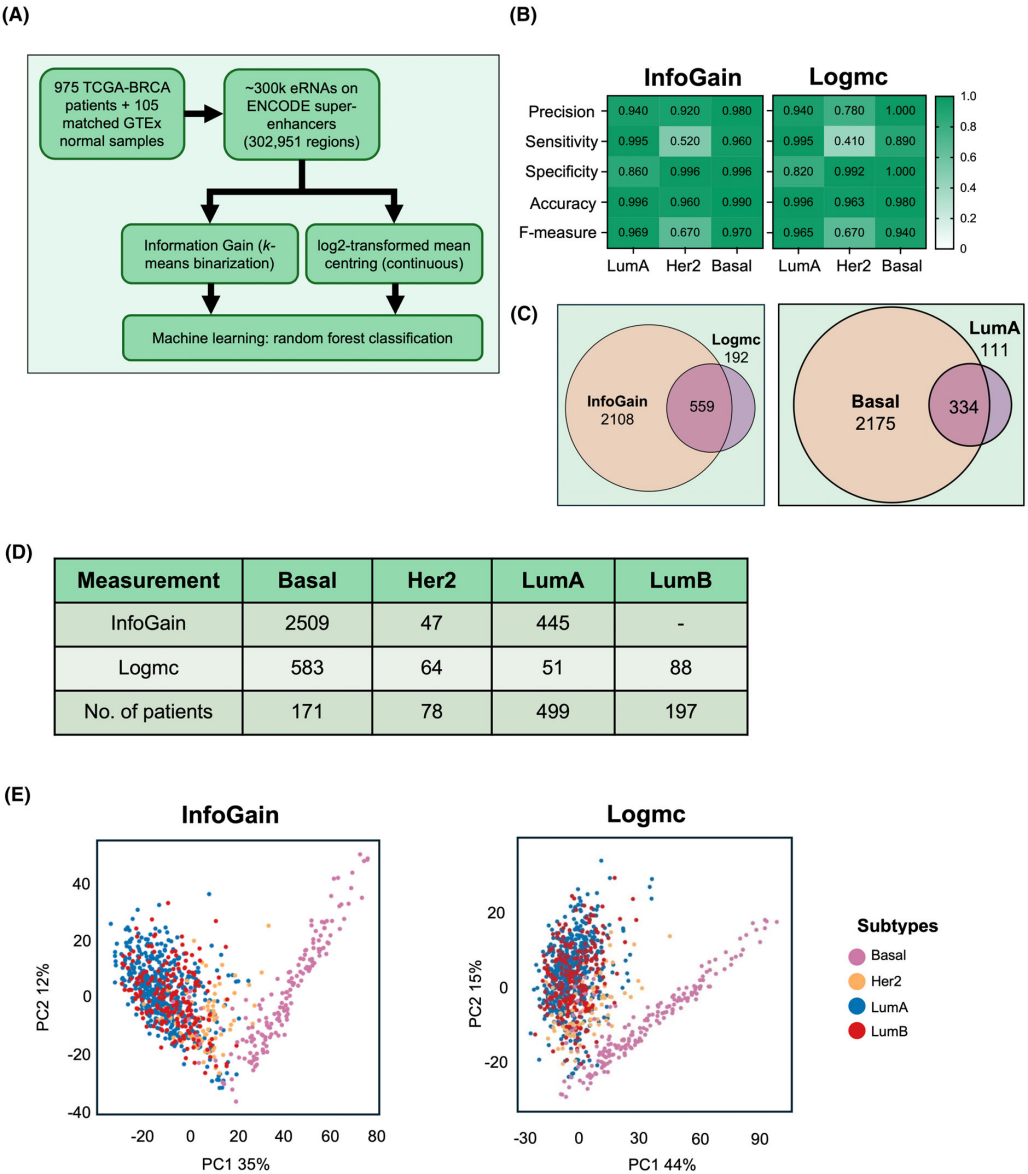
Machine learning models can efficiently identify subtype‐specific eRNAs. (A) Details of the analyses performed in this study. TCGA‐BRCA—Breast Cancer RNA‐seq data sets from The Cancer Genome Atlas (*n* = 975). 302 951 enhancer regions defined by ENCODE H3K27ac data sets were utilised. *k*‐means binning was used to convert the rpkm values to binarized values, and information gain values were calculated. Log2‐transformed centring of values to average expression (Logmc) provided continuous values. Machine learning‐based classification was performed using random forest. (B) Heatmap showing the statistics measures such as precision, sensitivity, specificity, accuracy, and F‐measure for information gain (InfoGain) and log2‐transformed mean centring (Logmc) measurements classifying each subtype (luminal A/B, Her2 and basal). (C) Venn diagram representing the overlap of all InfoGain and Logmc‐derived eRNA regions. (D) Number of eRNA regions classified per subtype with each measurement is shown in a table. Luminal B‐specific regions could not be identified with InfoGain measure. (E) PCA analysis showing the efficiency of InfoGain‐ and Logmc‐derived eRNssAs in classifying the clusters of patients from each subtype, using the top 2 principal components. LumA, luminal A; LumB, luminal B.

We generated two different measurements from the rpkm values of eRNA expression: (a) log mean centring (Logmc) and (b) information gain (InfoGain). Based on given eRNAs from both measures, we performed subtype‐specific classification using random forest to assess the efficiency of eRNAs in distinguishing between subtypes (Fig. 1A). We observed that the overall metrics evaluating the performance of the classification were slightly better for InfoGain‐identified eRNAs (Fig. 1B). However, sensitivity and F‐measure (which measures predictive performance) were poorer for Her2 eRNAs, partly due to the low number of Her2+ patients (*n* = 78). The InfoGain‐based approach identified a larger set of eRNAs, compared to that of Logmc (Fig. 1C), and 75% of Logmc eRNAs overlap with InfoGain eRNAs (Fig. S1A). Basal eRNAs were identified more than luminal eRNAs using both measures, and LumB‐specific eRNAs could only be identified with Logmc (Figs 1D and S1B). PCA and UMAP visualisations of the data based on subtype‐specific eRNAs showed a clear separation of basal and luminal subtypes using both measures, without further distinguishing LumA/B (Figs 1E and S1C). Both measurement‐derived Her2 eRNAs clustered their patients closer to luminal patients but in between luminal and basal patients. Surprisingly, the Logmc measure could not classify any distinct eRNAs for invasive ductal vs lobular cancer samples, and the InfoGain measure could identify only one lobular‐specific eRNA, which failed to cluster these histological subtypes (Fig. S1D).

**Fig. 2:**
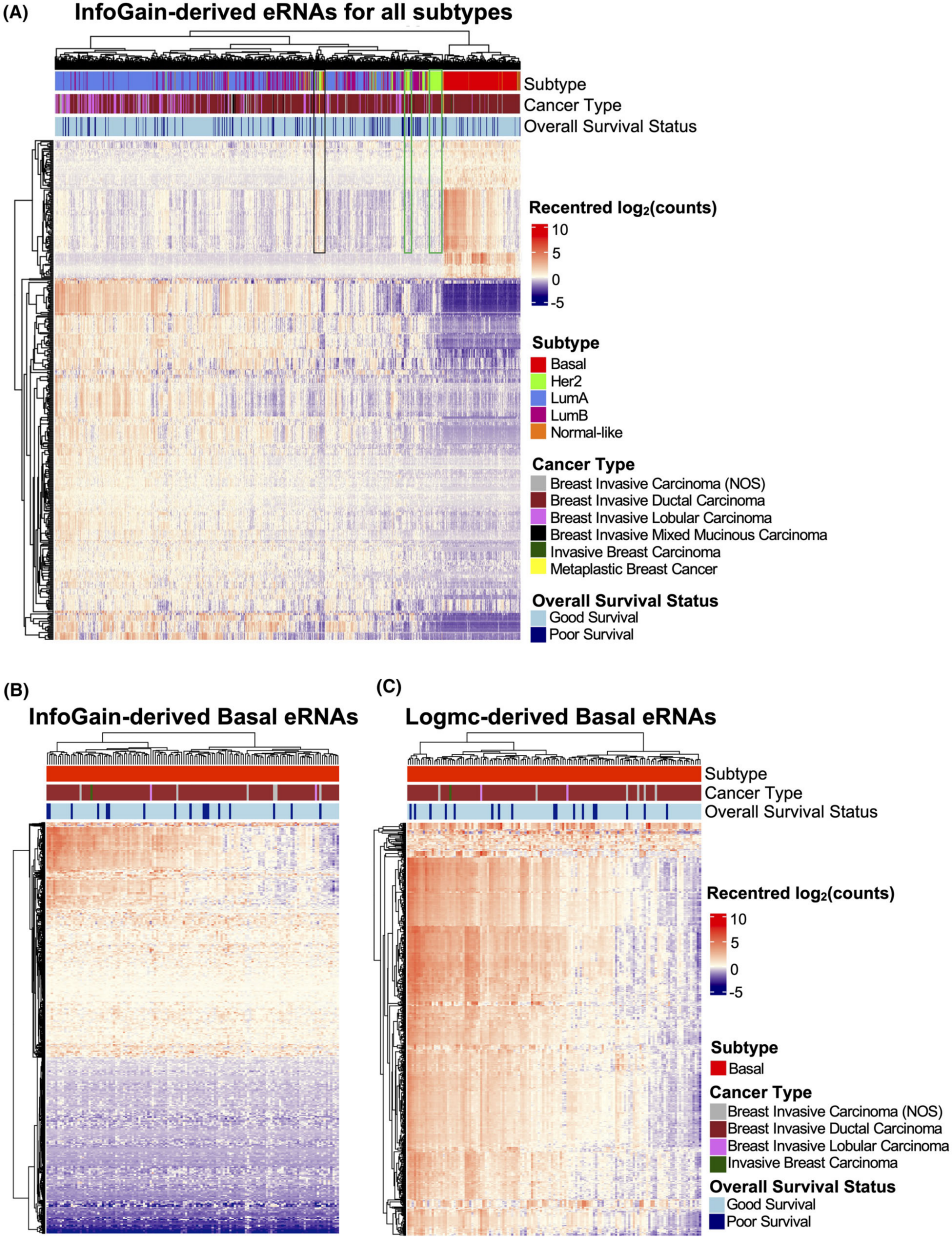
Heatmaps generated on subtype‐specific eRNAs classified using two different measures (InfoGain and log mean centring) showed better separation of subtypes. (A) Heatmap showing eRNA expression in log2‐transformed mean‐recentred values of breast cancer patient samples from TCGA on information gain (InfoGain)‐derived eRNA regions from all subtypes. Annotation of each patient with molecular subtypes, histological origin‐based cancer type (cancer type) and overall survival status is shown. Green rectangles highlight Her2 patients who show luminal‐like expression pattern and black rectangles highlight Her2 patients who show basal‐like expression pattern. (B, C) Heatmap showing eRNA expression in log2‐transformed mean‐recentred values of basal breast cancer patient samples for InfoGain (B) and log mean centring (Logmc) (C)‐derived eRNA regions of basal subtype. Annotation of each patient with molecular subtypes, cancer type and overall survival status is shown. LumA, luminal A; LumB, luminal B.

To visualise and verify the efficiency of the classification approach, we generated heatmaps with hierarchical clustering (Figs 2A and S2A). The majority of the basal subtype patients were clustered together based on InfoGain‐derived eRNAs, but Logmc‐derived eRNA‐based clustering produced two groups of basal patients. Highly expressed eRNAs in patients with the basal subtype had low expression in the luminal subtype and vice versa. InfoGain measure could clearly identify both high/low expressed basal eRNA regions with a similar expression pattern in most patients (Fig. 2B). However, all the Logmc‐derived basal eRNAs were highly expressed in most patients, but with mixed levels of expression in approximately 25% of patients (Fig. 2C). Hence, we could classify the InfoGain‐derived eRNAs in the basal subtype as high and low. Interestingly, basal high eRNAs showed low expression in luminal patients and vice versa (Fig. S2B,C). Hence, basal low eRNAs represent luminal‐specific eRNAs. While we observed a weak separation of high and low expressed InfoGain‐defined LumA eRNAs, we could not distinguish their expression levels as strong as that of basal‐specific eRNAs (Fig. S2D–G). Her2‐specific eRNAs were highly expressed in approximately two‐thirds of Her2+ patients (Fig. S2H–K). Even though our approach identified LumB eRNAs using the Logmc measure, the hierarchical clustering on the heatmap could not distinguish LumB from LumA (Fig. S2L), similar to the results in the PCA/UMAP plots (Figs 1E and S1C).

Altogether, both measurements classify eRNAs efficiently based on subtypes, but InfoGain allowed us to further distinguish samples based on high and low expression of eRNAs. InfoGain‐based classification also showed slightly better sensitivity for basal subtype and better precision and sensitivity for the Her2 patients, compared to Logmc. Hence, we focused on InfoGain‐defined eRNAs for further analyses.

### 3.2 Identification of subtype‐specific mRNAs to define proximally co‐expressed eRNA‐mRNA pairs (ProxCReAms)

**Fig. 3:**
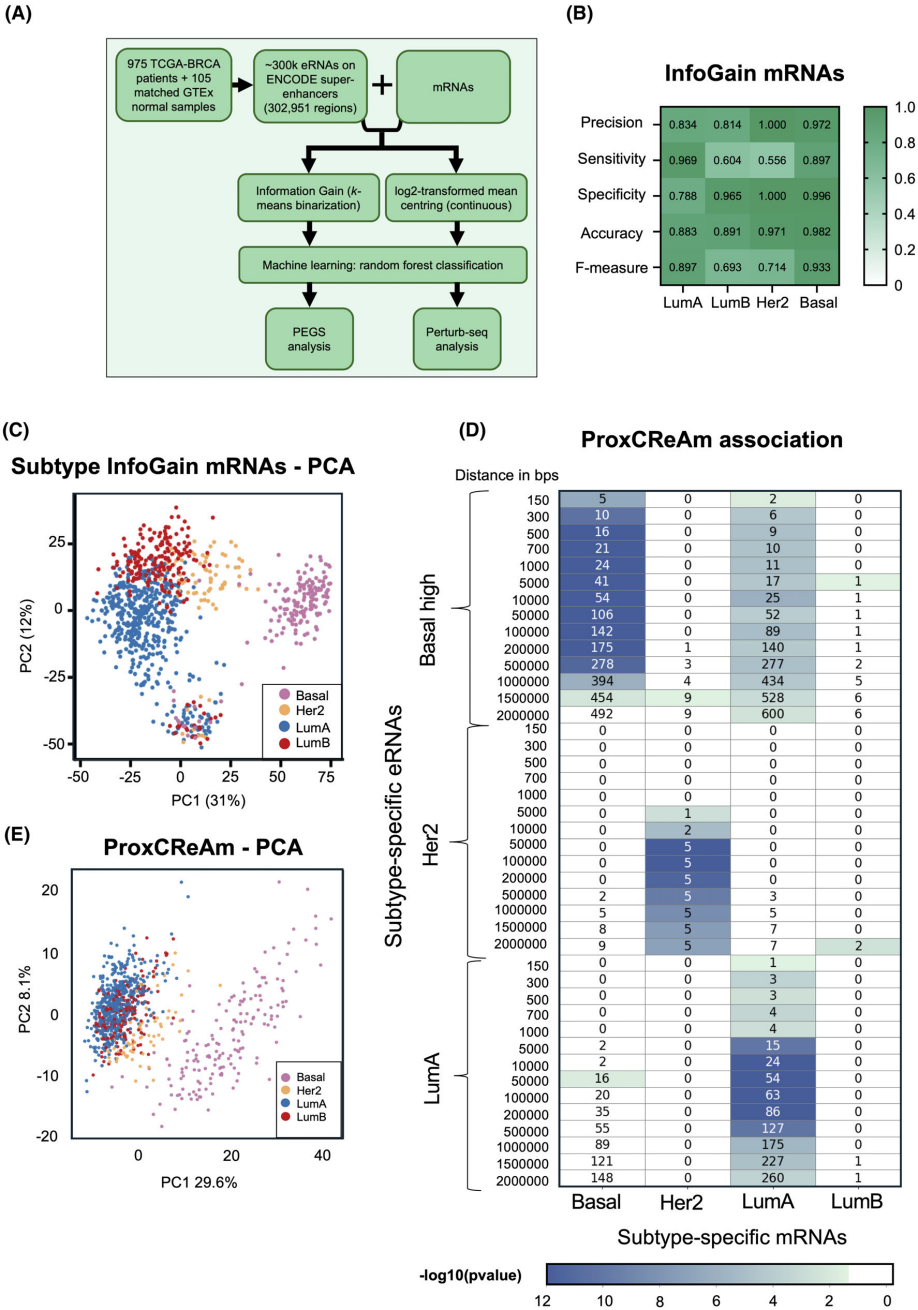
Association of subtype‐specific mRNAs with eRNAs helps in defining proximally co‐expressed eRNA‐mRNA pairs (ProxCReAms). (A) Details of the analyses performed to identify ProxCReAm eRNA‐mRNA pairs and to validate the functional relevance of these eRNAs by integrating with published Perturb‐seq data sets from breast cancer cell lines. (B) Heatmap showing the statistics measures such as precision, sensitivity, specificity, accuracy and F‐measure for information gain (InfoGain) measure classifying each subtype (luminal A/B, Her2 and basal) using mRNA expression datasets. (C) PCA analysis showing the efficiency of InfoGain‐derived mRNAs in classifying the clusters of patients from each subtype, using the top 2 principal components. (D) Heatmap showing the details of PEGS results to identify the ProxCReAm subtype‐specific eRNAs which are proximally associated with subtype‐specific mRNAs. The number represents the ProxCReAm mRNAs with different distances (from 150 bp to 2 MB) and the colour represents hypergeometric –log10 *P*‐value. (E) PCA analysis showing the efficiency of InfoGain‐derived ProxCReAm eRNAs in classifying the clusters of patients from each subtype, using the top 2 principal components. LumA, luminal A; LumB, luminal B; PCA, principal component analysis; ProxCReAm, Proximally Co‐expressed Regulatory eRNAs, which are Associated with mRNAs.

To understand if these subtype‐specific eRNAs are associated with specific mRNAs as downstream targets, we also employed our machine learning approach on coding regions with InfoGain measure to define subtype‐specific mRNAs (Fig. 3A). Sensitivity and *F*‐measure of LumB and Her2‐specific classification were poor (Fig. 3B). However, the InfoGain measure identified LumB‐specific mRNAs, but not eRNAs, and the metrics were slightly better for Her2‐subtype mRNAs than eRNAs. PCA, UMAP and visualisation with heatmaps showed better separation of Luminal A and B subtypes with the subtype‐specific mRNAs, compared to the eRNAs (Figs 3C and S3A–D); however, with one small cluster representing samples from different subtypes, separated by PC2, but it couldn't be resolved by PC3 which is limited to just 4% (Figs 3C and S3D). The mRNAs were clustered into two categories, similar to eRNAs (Fig. S3A). Even though clustered separately, most Her2 patients showed similar expression levels of mRNAs as basal patients. However, a significant proportion of Her2 patients showed a similar eRNA expression pattern as luminal patients (marked in green rectangles) and a small cluster of patients similar to basal subtype (Fig. 2A, marked in black rectangles). This suggests that the eRNA profiles capture the heterogeneity in Her2 patients efficiently.

To show if the subtype‐specific mRNAs are associated with proximal subtype‐specific eRNAs, we matched our InfoGain‐derived eRNAs and mRNAs using PEGS analysis (Fig. 3A) [29]. We defined these paired eRNAs as proximal co‐expressed regulatory eRNAs associated with mRNAs (ProxCReAms) and the proximal eRNA‐mRNA pairs as ProxCReAm pairs. We performed this analysis across genomic distances from 150 bp to 2 Mb (Fig. 3D). Highly significant associations were observed between 150 bp and 1 Mb for basal ProxCReAm pairs and 5000 bp–1.5 Mb for luminal A pairs. For further analysis, we selected the ProxCReAm pairs within a 1 Mb distance, as their associations were strongly significant in all subtypes and consistent with other tools, such as GREAT, and other published analyses [59, 60]. Overall, 81.45% of the subtype‐specific eRNAs were associated with subtype‐specific mRNAs within 1 Mb distance, that is, 79.9% of basal high eRNAs (*n* = 1145) with 394 subtype‐specific mRNAs, 84.9% luminal A eRNAs (*n* = 378) with 175 mRNAs and 95.7% of the Her2 eRNAs (*n* = 45) with five mRNAs. While ProxCReAm eRNAs showed heterogeneity within groups and similar profiles as the subtype‐specific eRNAs (Figs 3E, S3B and S3E), their expression patterns grouped some of the luminal B or lobular cancers together (Fig. S3B).

To investigate the clusters observed in lobular cancers using information gain (Fig. S3B), we further explored the RNA expression of ductal versus lobular cancers. We classified mRNAs to distinguish ductal and lobular‐specific gene expression. We detected just 38 specific mRNAs using information gain with a standard cutoff of 0.05; however, the separation was better (Fig. S3F,G). With this threshold, only one eRNA was detected. Hence, we used a low cutoff for information gain (above 0.01), which showed 7440 eRNAs (Fig. S3H) and then performed the PEGS analysis on these eRNAs. We observed that only 223 ProxCReAm lobular‐specific eRNAs were associated with 33 mRNAs within 1 MB distance (Fig. S3I). Further analyses of these ProxCReAm eRNAs could classify ductal versus lobular cancers (Fig. S3J,K) slightly better than eRNAs alone (Fig. S3H). Pathway analyses of the 33 ProxCReAm ductal/lobular‐specific mRNAs showed enrichment of genes associated with chromosome 16q loss [61], genes upregulated in peripheral zone of the tumour compared to the central zone [62] or anaplastic/poorly differentiated conditions [63], etc. (Fig. S3L). 16q lost region includes tumour suppressors like *CDH1*, which are frequently deleted in lobular breast cancers and its loss is strongly associated with the invasive nature of cells due to the lack of epithelial–mesenchymal transition [64]. Peripheral zone‐upregulated genes include mitotic M‐phase‐related genes such as *NCAPG, TUBA1C, and PSMD14* [65]. Overall, even with the low threshold eRNAs showing less variation, ProxCReAm eRNAs could classify the lobular subtypes better than the higher threshold eRNAs, and a significant number of them could be integrated with the mRNAs associated with aggressive pathways relevant to lobular cancers.

Hence, our analyses defined proximally co‐regulated eRNAs with their downstream mRNAs in a subtype‐specific manner for the first time in breast cancers, and this approach separates the histological subtypes more efficiently than the selected eRNAs.

### 3.3 ProxCReAm eRNAs are associated with subtype‐specific gene pathways

**Fig. 4:**
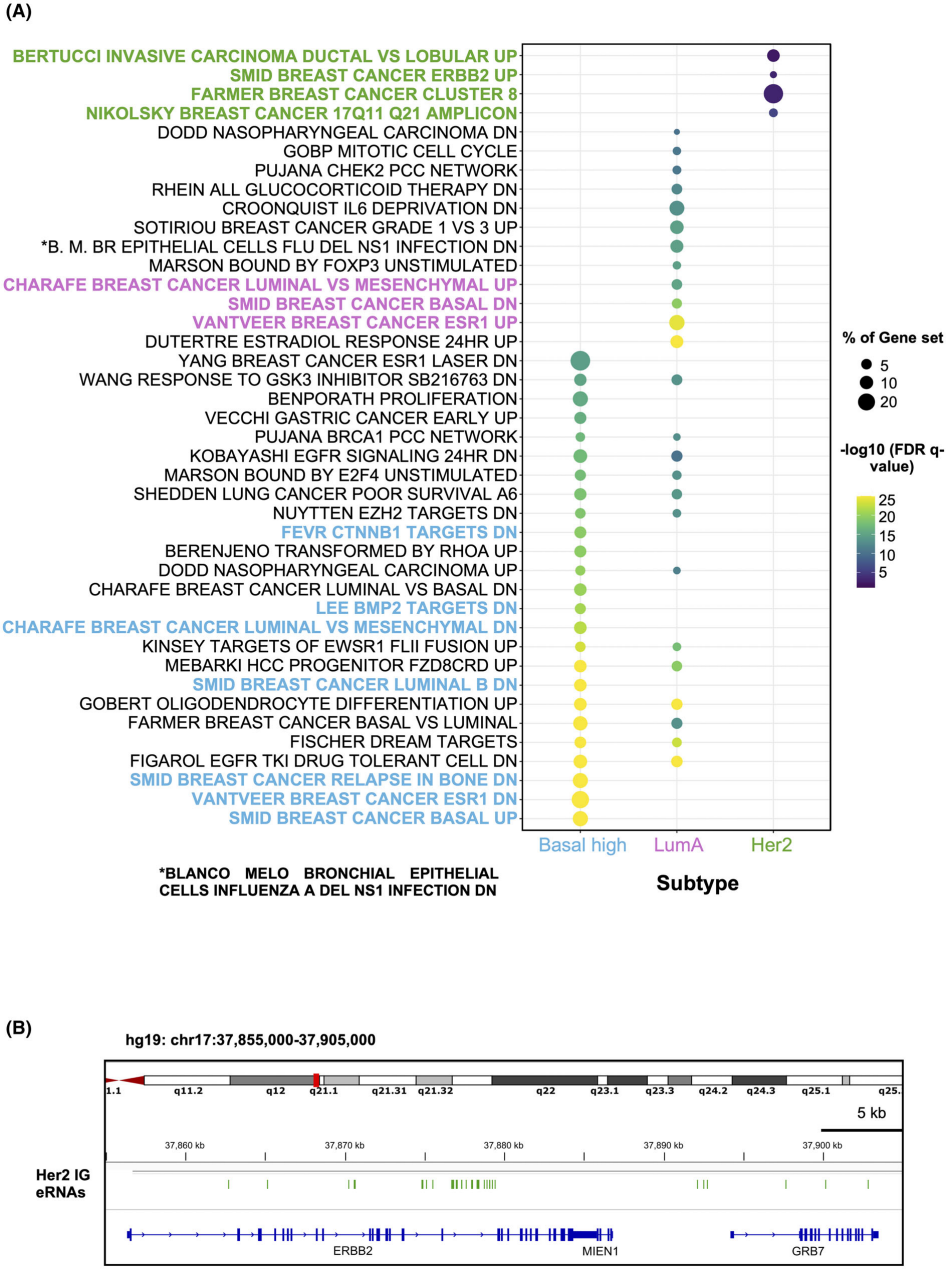
ProxCReAm eRNAs are associated with subtype‐specific gene pathways. (A) Dot plot showing gene pathway analyses performed on InfoGain‐derived ProxCReAm mRNAs of (Basal high) in basal subtype, luminal A (LumA) and Her2‐specific regions using MSigDB database (CGP, C5 and C6 gene sets). Colour keys represent −log10 FDR q‐value and percentage of gene set (percentage of the ratio of overlapped genes by total number of genes in a pathway gene set). Pathway names were highlighted in blue (basal high), pink (luminal) and green (Her2) specific to subtypes. Only significant pathways are shown. Pathways specific to luminal, ER‐regulated, basal, Her2, β‐catenin, BMP2 and relapse‐related signatures are highlighted. Pathways were sorted and visualised using −log10 FDR q‐value per subtype. (B) Multiple gene view around q11‐21 amplified region from chromosome 17 close to *ERBB2* (Her2) gene with the Her2‐specific eRNA regions derived from InfoGain (green). CGP, chemical and genetic perturbations; IG, InfoGain; ProxCReAm, Proximally Co‐expressed Regulatory eRNAs, which are Associated with mRNAs.

To verify the biological importance of the ProxCReAm eRNAs per subtype, we examined their associated mRNAs for pathway enrichment with annotated pathways from GSEA – Molecular Signature database. We observed that basal high eRNAs were associated with basal‐specific pathways or luminal/ER downregulated pathways and vice versa (Fig. 4A). Luminal eRNAs identified ER/luminal upregulated or basal downregulated pathways. Bone relapse and Wnt‐signalling (β‐catenin and BMP2)‐specific downregulated genes were also associated with basal high eRNAs.

Many ER target/luminal genes (*ESR1*, *MLPH*, *CT62/THSD4, XBP1*) enriched in these pathways are lineage‐specific factors reportedly upregulated in ER+ breast cancers [66, 67, 68]. To verify if the eRNA loci close to these genes are active and bound/regulated by ER in patients, we examined ER binding using published ER ChIP‐sequencing data sets from drug responsive (MCF7, ZR‐75‐1) and resistant (BT474, MCF7 TamR Tamoxifen resistant) ER+ cell lines, primary patient samples with differential survival status (good and poor outcome) and metastatic samples (Fig. S4) [19]. We also visualised ATAC‐seq data sets from ER+ patients in the TCGA cohort, H3K27ac ChIP‐seq data sets from MCF7 and ZR‐75‐1, as well as CAGE and GRO‐seq data from MCF7, to associate the identified eRNA regions with chromatin accessibility and bidirectional transcription. We observed that the subtype‐specific eRNAs are strongly marked with ATAC‐seq, H3K27ac, CAGE and GRO‐seq signals thus representing active bidirectional transcription (Fig. S4). All the ER binding sites are accompanied by open and active enhancer marks with bidirectional transcription (either GRO‐ or CAGE‐positive) but they are not on the exact location of subtype‐specific eRNA loci. For example, eRNA regions close to genes *ESR1* and *THSD4* are 250–1000 bps away from the centre of the ER binding sites (Fig. S4A,B). The eRNA regions close to gene *MLPH* possess both active bidirectional transcribing ER‐bound sites close or distal to the selected eRNA loci (around 1.5 kb or more) and ER‐unbound sites (Fig. S4C). These distal ER binding sites of *MLPH* and *XBP1* (Fig. S4D) are close to the regions from the list of 300 K eRNA loci and they were simply not identified as subtype‐specific regions. Hence, our results suggest that the occupancy of ER might not be present on all subtype‐specific eRNA loci, but our subtype‐specific eRNA sites are representative of bidirectional transcription.

Her2‐specific ProxCReAm eRNAs showed enrichment of pathways related to *ERBB2* (gene of Her2) (Fig. 4A). These genes also showed significant association with ductal‐specific upregulated genes, compared to lobular cancers. The pathways, ‘NIKOLSKY_BREAST_CANCER_17Q11_Q21_AMPLICON’ [69] and ‘FARMER_BREAST_CANCER_CLUSTER_8’ [70] represent genes from chromosome region 17q11‐21, and part of this region harbours the genes *ERBB2* and *MIEN1* which are activated/amplified in Her2‐positive cancers (Fig. 4B). There are 30 Her2‐specific eRNA regions in the vicinity of these genes. Integrating these eRNAs with published HiC datasets in two different Her2‐enriched cell lines (HCC1954 and BT474) suggests that these eRNA regions constitute within a single or multiple topologically associated domains (TADs) and form 3D chromatin loops, which are characteristic of highly active enhancer regions (Fig. S4E,F). 3D analysis also shows that another TAD overlapping with these eRNA regions and other eRNA regions potentially trans‐regulating a different set of genes such as *ZPBP2*. This suggests that our analysis identified relevant eRNA loci for Her2‐type breast cancers, but caution should be employed to rule out any possible patient‐specific copy number amplifications confounding the eRNA expression levels. Altogether, our pathway analyses suggest that eRNA expression can be associated directly with active, open bidirectional transcribing regions, key 3D chromatin interactome networks and thus, regulating subtype/lineage‐specific gene pathways.

### 3.4 Subtype‐specific eRNAs are associated with key transcription factors and epigenetic regulators

**Fig. 5:**
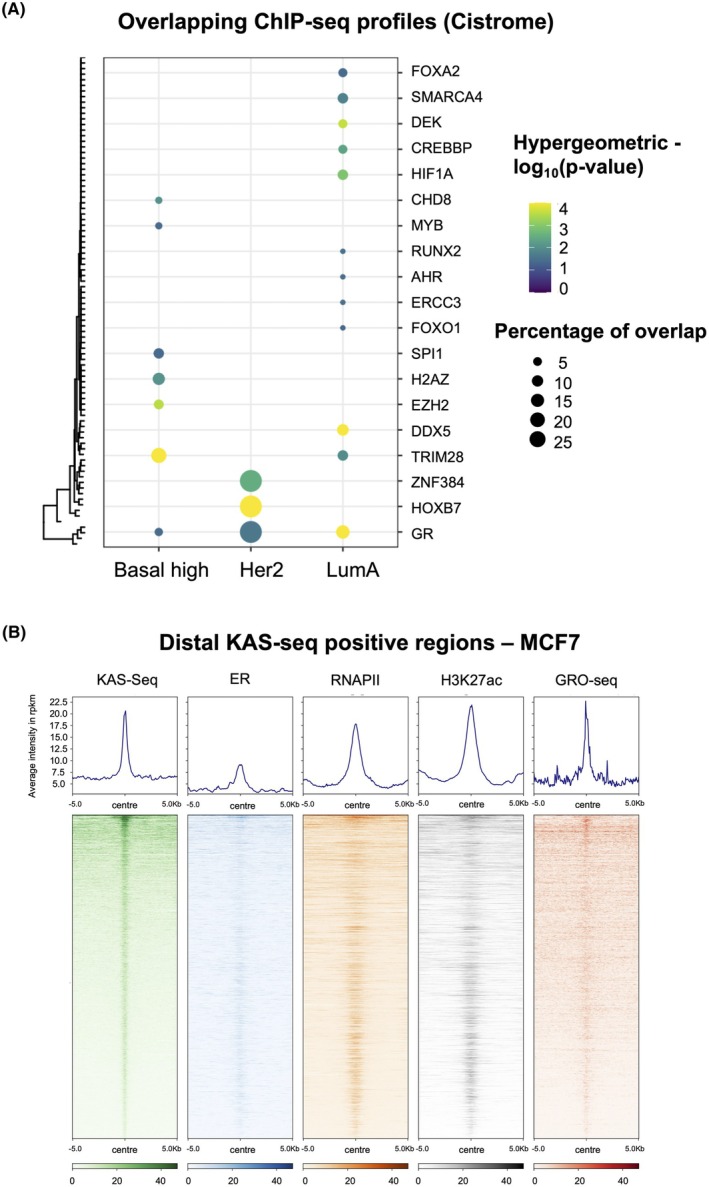
Subtype‐specific eRNAs are associated with key transcription factors and epigenetic regulators. (A) Dot plot showing Cistrome‐based overlap of binding with published ChIP‐seq data sets performed on InfoGain‐derived ProxCReAm eRNA regions highly expressed in basal subtype (Basal high), luminal A (LumA) and Her2‐specific regions. Colour keys represent hypergeometric −log10 *P*‐value calculated by phyper function from hypergeometric distribution (hypergeometric package) from R and percentage of overlap of the eRNA regions with the published binding sites for each factor. Only significant factors are shown. (B) Average density plots and heatmaps showing the rpkm values of eRNA expression from GRO‐seq from 40 min of oestrogen‐treated MCF7 cells (red) and KAS‐sequencing (green), and ER, RNA polymerase II, and H3K27ac occupancy from MCF7 cells which are continuously grown with serum with hormones (blue, orange and grey, respectively). The regions are mapped ±5 kb away from the centre of distal eRNA peaks (8589 regions) defined by KAS‐seq in MCF7 cells, with blacklist and sequencing artefacts subtracted. ER, oestrogen receptor; GRO‐seq, global run‐on sequencing; InfoGain, information gain; KAS‐seq, kethoxal‐assisted single‐stranded DNA sequencing; ProxCReAm, Proximally Co‐expressed Regulatory eRNAs, which are Associated with mRNAs; RNAPII, RNA polymerase II.

To identify the important factors associated with the ProxCReAm eRNAs, we integrated them with the published ChIP‐seq data sets of transcription factors and epigenetic regulators from Cistrome platform [17] (Fig. 5A). Basal high regions were associated distinctly with Tripartite motif‐containing transcriptional corepressor TRIM28 and histone variant H2AZ which usually marks active enhancers, and the Polycomb Repressive complex‐2 subunit EZH2 which promotes the deposition of gene repressive mark H3K27me3. We also observed enrichment of oncogenic factors, which are upregulated in basal‐like or TN breast cancers, such as Spi‐1 protooncogene (SPI1), MYB and chromodomain‐helicase DNA binding protein (CHD8) [71, 72, 73]. LumA subtype eRNA loci showed significant enrichment of nuclear receptors such as glucocorticoid receptor (GR) [74] and aryl hydrocarbon receptor (AHR) [75], well‐known ER coactivators SWI/SNF‐related BAF chromatin remodelling complex subunit ATPase‐4 (SMARCA4, a major subunit of SWI/SNF chromatin remodelling complex) [18, 76], cyclic AMP response element‐binding protein (CREBBP) [77] and hypoxia‐inducible factor 1 alpha (HIF1A). As consistent with ER not being bound exactly on eRNA loci (Fig. S4), ER was not significantly enriched in LumA regions, but forkhead domain‐containing proteins FOXA2 and FOXO1, which bind to the same motif of luminal A‐specific FOXA1 pioneer factor that opens the chromatin for ER binding [78]. Her2 subtype regions showed strong enrichment of several transcriptional regulators including GR, a coactivator of *ERBB2* gene, HOX cluster protein HOXB7 [79] and zinc finger protein ZNF384.

Furthermore, to look at the frequency of DNA motifs associated with transcription factors on the selected eRNA loci, we integrated the subtype‐specific eRNAs with the 1000 bp flanked regions of the TCGA‐BRCA‐based ATAC‐seq sites, which represent accessible DNA regions, where transcription factors bind (Fig. S5A,B). 22.7%–47% of the eRNA regions overlapped with the corresponding ATAC‐seq sites, with one eRNA matching with multiple ATAC‐seq sites. As the integrated sites were low in number, motif enrichment analyses were performed on all subtype‐specific eRNAs. We utilised the basal and luminal‐specific eRNA‐integrated ATAC‐seq sites after excluding the regions which align with first introns and promoters. Due to fewer regions, Her2 sites did not overlap with the ATAC‐seq sites. Consistent with the results from Cistrome analyses, we could not observe any direct motifs of ER or steroid hormone receptors for LumA regions. However, FOX motifs were more enriched in LumA regions than basal high regions. Combined motifs of Ets‐related and forkhead domain factors were enriched in LumA regions, consistent with the coactivator role of Ets1 and forkhead domain proteins in ER+ signalling [80]. The binding motifs of retinoic acid receptor RAR motif were enriched in Basal high regions. Moreover, motifs of regulators of differentiation (MADS box factors, MEF2A/C), AP1 (Jun‐related), POU domain factors, STAT, NF‐κB and homeodomain‐containing factors HOX were more enriched in basal regions. Overall, Cistrome binding overlap and motif analyses on eRNA loci suggest key transcription factors and epigenetic modulators specific to each subtype, more specifically, the association of nuclear receptors other than the oestrogen receptor or forkhead domain proteins with luminal eRNAs/enhancers.

To validate the ER‐independent nature of LumA eRNAs, we performed enhancer RNA profiling using kethoxal‐assisted ssDNA sequencing (KAS‐seq [32]) in the MCF7 cell line, which is an ER+ LumA type breast cancer cell line. This assay identifies regions of single‐stranded DNA (ssDNA) representing active transcription. We profiled the KAS‐seq signals on distal sites 5 kb away from genes (8589 distal KAS‐seq‐positive regions) and integrated them with GRO‐seq to detect nascent RNA [4] and ER, RNA polymerase II (RNAPII), and H3K27ac occupancy (Fig. 5B). We observed that ER binding is weak on the top ssDNA and GRO‐seq‐positive sites which are bidirectionally transcribing. Overall, our findings suggest that not all the actively bidirectionally transcribing distal eRNA loci possess ER binding.

### 3.5 InfoGain‐derived eRNAs can be prognostic in breast cancers

**Fig. 6:**
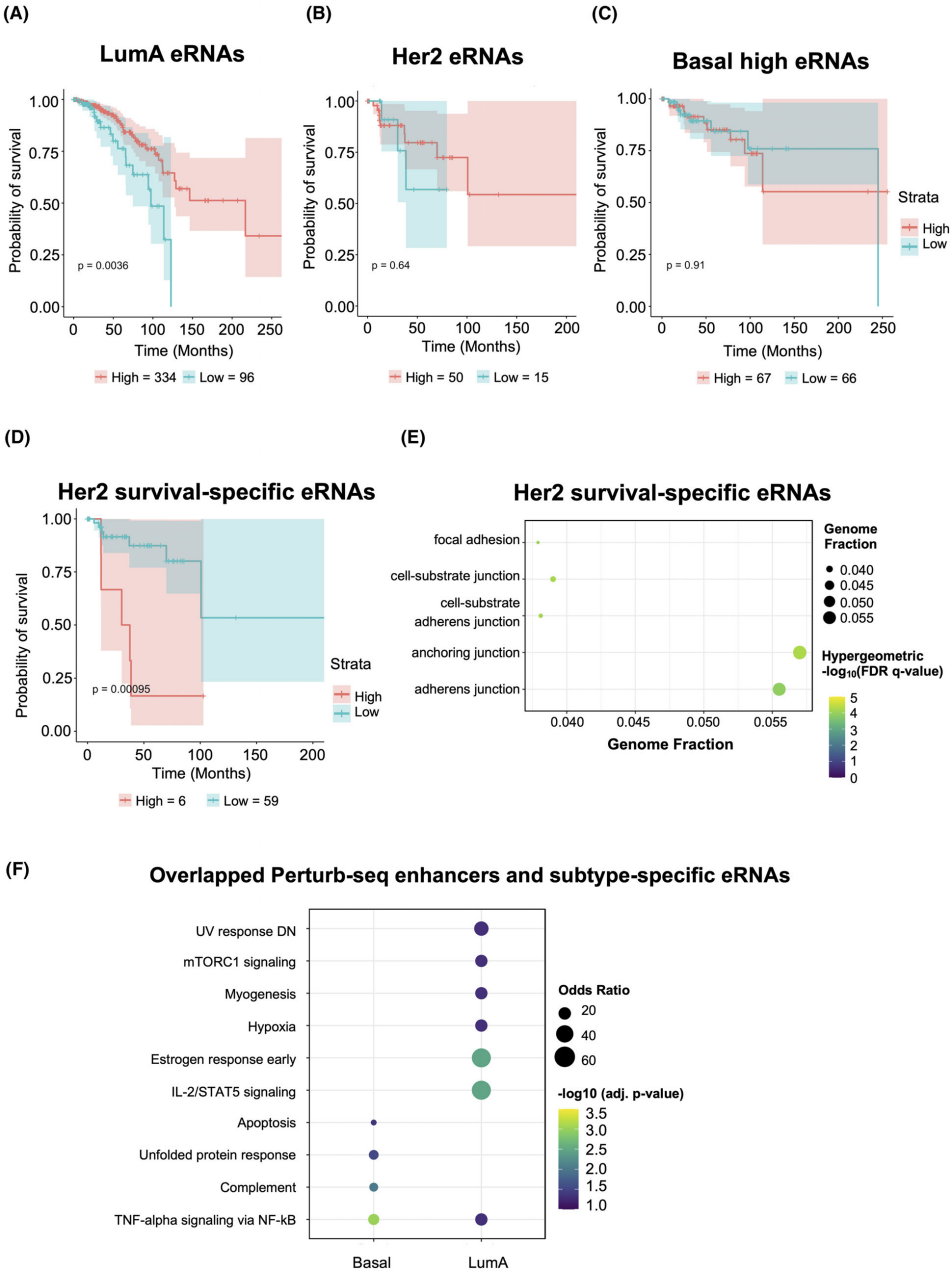
InfoGain‐derived eRNAs can be prognostic and functionally relevant in breast cancers. (A–C) Kaplan–Meier plots showing the probability of overall survival of patients with each subtype (luminal (A), Her2 (B) and basal high (C) eRNAs) who show low and high average expression of InfoGain‐derived subtype‐specific eRNAs among patients. Number of months of patient survival after diagnosis is shown. Plots represent 430 LumA patients, 65 Her2 patients, and 133 Basal patients. *P*‐values were calculated using log‐rank test. Numbers of patients in each category (high and low average eRNA expression) are mentioned under each plot. (D) Kaplan–Meier plot showing the probability of overall survival of Her2‐positive patients who show low and high average expression of InfoGain‐derived Her2 subtype‐specific eRNAs among patients, classified based on survival (dead‐ poor survival or alive‐ good survival). Number of months of patient survival after diagnosis is shown. *P*‐value was calculated using log‐rank test. Numbers of patients in each category (high and low average eRNA expression) are mentioned under each plot. (E) Dot plot representing pathway enrichment analysis on Her2‐specific InfoGain‐derived eRNAs showing significant association with cell adhesion pathways. Colour keys represent hypergeometric −log10 FDR q‐value and genome fraction (ratio of observed genes by total number of genes in a pathway gene set). Only significant pathways are shown. (F) Dot plot representing pathway enrichment analysis on the overlapped enhancer regions from Perturb‐seq datasets (ER‐ and ER+ LumB cell lines) with the subtype‐specific InfoGain‐derived eRNA regions from Basal and LumA subtypes, respectively. As InfoGain did not classify the LumB eRNAs due to the high similarity with LumA subtype, LumA eRNAs were overlapped. Colour keys represent −log10 adjusted *P*‐value and Odds ratio. Only significant pathways are shown. LumA, luminal A; LumB, luminal B; InfoGain, information gain.

Next, we investigated the prognostic power of subtype‐specific eRNAs. As ProxCReAm eRNAs are low in number, we focused on all the subtype‐specific eRNAs. Kaplan–Meier curve‐based survival analysis of LumA‐specific eRNAs showed that patients with high expression of these eRNAs have better survival (Fig. 6A). This is consistent with the expression of ER and its target genes being positively associated with survival in luminal patients [81]. Expression of Her2 and basal subtype‐specific eRNAs did not show any significant association with patient survival (Fig. 6B,C).

To identify subtype‐specific prognostic eRNAs with high confidence, we performed random forest classification on both measures, distinguishing patients with good or poor outcomes per subtype. The InfoGain measure successfully identified 342 eRNAs, which classified the survival status of the Her2 subtype with a 0.1 threshold (Fig. S6A,B), but not for other subtypes, even with a less stringent threshold. Survival and hierarchical clustering analyses showed that Her2‐positive patients with high average expression have worse outcomes (Figs 6D and S6A). The genes close to these prognostic Her2 eRNAs are heavily enriched in cell adhesion/junction pathways (Fig. 6E), implying the importance of deregulation in extracellular matrix or epithelial–mesenchymal transition pathways in poor prognosis. Logmc measure could not identify any eRNAs specific to survival.

### 3.6 Functional validation of the subtype/survival‐specific eRNAs

To strengthen the functional relevance of the subtype/survival‐specific eRNAs, we integrated our selected eRNAs with published Perturb‐seq data sets [24]. These were performed after CRISPRi‐based perturbation of 3513 candidate enhancers and mRNA promoters, followed by single‐cell RNA‐seq in ER+ and ER‐ breast cancer cell lines to measure the effect of these elements on local and global transcription. Despite the limited number of enhancers examined in this published work, 8 of our subtype‐specific eRNAs and 21 survival‐specific eRNAs overlapped with the published perturbed enhancers, which have been shown to affect gene expression. Moreover, these integrated LumA‐specific eRNAs were strongly enriched with IL‐2/STAT5 signalling, which drives metastasis in ER+ breast cancers [82], oestrogen response signalling, etc. (Fig. 6F). Basal eRNAs showed enrichment of TNF‐α and Complement response, which is consistent with the strong immune regulation observed in ER‐tumours [83] and apoptosis‐related pathways. A gene regulated by these Perturb‐seq overlapped survival‐specific eRNAs, *EMID1*, which promotes cell proliferation and metastasis and has a strong proximal LumB‐associated perturbed enhancer (Perturb‐seq results of *EMID1* expression upon CRISPRi of the enhancer: −log10 *P* = 2.357, log CPM = −0.591), is highly expressed in breast cancer tissues compared to normal tissues and is associated with better survival in breast cancer [84, 85]. Altogether, our findings strongly suggest that our selected eRNAs are functionally relevant with lineage‐defining functions by associating with the gene expression of subtype/survival‐specific mRNAs.

## 4 Discussion

Our studies attempted to identify eRNAs which are specifically expressed in each subtype based on gene expression, anatomical origin and survival in breast cancer. Our machine learning approach can identify subtype‐specific eRNAs and luminal and Her2‐specific prognostic eRNAs, even though eRNA expression in cancers of LumA and LumB subtypes looks majorly similar. Our integrated analyses with proximal subtype‐specific mRNAs, ATAC‐seq sites and further validation with Perturb‐seq datasets identified ProxCReAm eRNAs, which have functional activity and biological and clinical significance, and the key transcription factors and epigenetic proteins which can play important roles in lineage‐specific breast cancers. Interestingly, our integrated analyses using ProxCReAm eRNAs can distinguish lobular cancers efficiently. Furthermore, our study identified nuclear receptors other than ER and pioneer factors which are important in breast cancers. Hence, our study supports the avenue of utilising eRNAs as lineage‐specific biomarkers for identifying upstream regulators and prognosis.

Using eRNA expression datasets as a tool, our goal is to define relevant transcriptional regulators and verify whether their association with prognosis is improved by subtyping. Hence, we focused on identifying subtype‐specific eRNAs using machine learning tools. We showed that eRNAs can associate with subtypes using two different tools, where the InfoGain measure can identify both low‐ and high‐expressed subtype‐specific eRNAs. As the molecular subtypes in breast cancers were initially defined using gene expression data sets, mRNAs performed better as predictors of subtypes. Interestingly, our selected eRNAs and the ProxCReAm pairs could classify the tumour heterogeneity and lobular subtypes, which were hard to distinguish. This highlights the role of epigenetic regulation and enhancer activity influencing these cancer processes, hence determining the dynamic changes in these systems would be beneficial. Moreover, identifying regulatory networks with emphasis on transcription factor binding motif analyses is not achievable using mRNA data sets. In our study, analyses of the eRNA loci with appropriate flanks, regardless of their integration with active enhancer regions, provided a high‐resolution landscape of TF and epigenetic networks. These sorts of analyses usually require ATAC‐seq or H3K27ac data sets, but these assays need fresh frozen tissue material and laborious experimental designs compared to RNA‐seq data sets. Furthermore, eRNA‐transcribing enhancers represent highly active enhancers, while ATAC and H3K27ac data sets can identify all enhancers which can be inactive or poised, but captured due to the dynamic nature of enhancers. We demonstrate that traditional RNA‐seq data sets mapped on active enhancer regions showing eRNA transcription would be sufficient to identify the highly active TF network and gene‐enhancer regulatory frameworks in a subtype‐specific manner, hence emphasising the potential of eRNA studies. The findings from this study would be beneficial for molecular biologists to know how enhancer transcription can be associated with gene regulation through deregulated transcription factor networks in patients.

Around 90% of eRNAs are bidirectional and nonpolyadenylated [86]. However, TCGA expression data sets are based on polyA‐enriched RNA‐seq assays, which capture only polyadenylated RNAs. Thus, analysing the expression of eRNAs on mRNA‐seq data sets might not be adequate. For this study, we utilised pan‐cancer‐based published eRNA atlas datasets from Chen *et al*. 2018 and 2020, where the abundant RNA signals on intronic and intergenic regions are included, and TSS/exon‐based signals are excluded (Fig. S6C). These studies utilise the advantage of identifying eRNAs on a large sample size; hence, the mRNA signals are negligible. Furthermore, nonpolyadenylated transcripts are highly unstable leading to a low half‐life of 2–3 min for eRNAs [87]. Hence, detection of polyadenylated eRNAs on highly expressed enhancer regions can be easily adapted to any clinical lab for routine diagnostic purposes on FFPE samples where RNAs are heavily degraded. Further work on eRNA expression on patient samples using robust nascent RNA detection methods like KAS‐seq or ChRO‐seq is still warranted to acquire the strongest lineage‐specific or prognostic eRNAs. However, this is a tedious process, and currently, there are no nascent RNA data sets available on breast cancer patient samples.

Our study identified similar subtype‐specific eRNAs from different measures. To note, InfoGain‐based analyses identified both high/low expressed and prognostic eRNAs. InfoGain measure with the approach of binarisation with *k*‐means (*k* = 2) has the potential to capture both strongly expressed eRNAs, which are differential between subtypes, as well as low expressed sparser on and off eRNAs. In the first case, although eRNA is highly expressed in all patients, the higher expression mode becomes 1 and the lower expressed mode becomes 0. However, in the case of low expression with on and off types of expression, recentred Logmc would not generate a strikingly high value. Furthermore, binarisation is also helpful in performing better clustering and classification, as distinguishing between data points becomes more effective. Hence, using InfoGain for machine learning‐based classification of biomarkers would provide greater benefit. This approach can also be used to identify prognostic biomarkers in other cancers, where their heterogeneous nature is established.

We identified that lobular and ductal cancers are indistinct in eRNA expression, which is similar to previous studies with mRNA expression [88, 89], and however, the integration of low threshold eRNAs with mRNA candidates separated these subtypes efficiently by identifying key eRNA‐mRNA pairs such as *CDH1* or its proximal genes/enhancers, which is lost in around 65% lobular cancer patients [64] and networks associated with aggressiveness [62, 63, 65]. This suggests that the enhancer activation/reprogramming in lobular cancers might drive aggressiveness and drug resistance against existing ER‐targeted therapies like tamoxifen. Hence, understanding these mechanisms is crucial to develop lobular‐specific drug targets. Various studies established that the tumour and immune microenvironment in lobular cancers are distinct in comparison to ductal cancers [90, 91]. This emphasises the utility of single‐cell RNA‐seq data sets performed on high stroma vs tumour content to be more appropriate for identifying better molecular signatures in lobular cancers.

Our integrative analyses with bidirectional transcripts observed in GRO‐seq and CAGE datasets suggested that the lack of association of ER on exact eRNA loci is not due to analysing 1D polyadenylated eRNA transcripts. ER motifs or overlap with ER binding could not be observed on flanked regions of luminal eRNAs and this observation is supported by the distribution of ER binding at least 250–1000 bps away close to eRNA loci. Interestingly, these regions showed strong enrichment of other nuclear receptors like GR, AHR and forkhead transcription factors. We identified eRNAs close to *ESR1* and other lineage‐determining genes with strong distal ER binding in breast cancer patient samples. Altogether, this suggests that nuclear receptors other than ER coupled with the forkhead family pioneer factors can play key roles on active enhancers in primary ER+ tumour samples. Furthermore, it is possible that ER can associate with these factors distally. Interestingly, basal‐specific eRNAs identified the involvement of the corepressor TRIM28 which promotes cancer stem cell proliferation and metastasis in triple‐negative breast cancers [92, 93]. Importantly, our results suggest that GR and RAR nuclear receptors may associate with AP1 pioneer factors in basal tumours. GR activation is previously shown to promote metastasis in triple‐negative breast tumours [94]. Hence, our integrated eRNA analyses provide a promising avenue of identifying functionally relevant transcription factor networks which can be targetable.

In the previous studies, Chen *et al*. [13, 14] identified 326 prognostic eRNAs in breast cancers without subtyping them. Surprisingly, these eRNAs do not overlap much with any eRNAs classified by our approaches and they show a weak association with survival (Fig. S6D). Our InfoGain‐based analyses showed that eRNA expression can be associated with good or poor prognosis dependent on subtype. While eRNA expression is associated as a positive biomarker for therapeutic response to BET inhibitors and immune checkpoint inhibitors [13, 95], high expression of genomic instability‐specific eRNAs is associated with worse survival in breast cancer patients [96]. Moreover, in our study, many of the eRNA‐overlapped enhancer loci could be functionally validated to show a strong effect on gene expression and their target mRNAs regulate proliferation, metastasis and predict patient survival. Hence, our study underscores the importance of molecular subtyping before identifying any prognostic biomarkers in heterogeneous cancers. Further work on RNA‐seq data sets with an adequate number of patients across minor subtypes would be beneficial.

## 5 Conclusions

Altogether, our study emphasises the importance of subtyping to identify functionally relevant prognostic eRNAs/enhancers, molecular signatures and upstream regulators which can be therapeutically relevant and targetable. Our findings further highlight the possibility of constructing gene regulatory transcription factor networks related to patient survival by looking into classical unidirectionally transcribed polyadenylated RNA‐seq data sets, as other laborious epigenetic analyses require patient samples in fresh frozen conditions which are extremely limited. Further validation of these enhancer regions in relevant preclinical models would be beneficial in understanding their biological insights and clinical importance.

## Conflict of interest

The authors declare no conflict of interest.

## Author contributions

SN conceptualised the project. AYP, PZ, MI and SN designed the project; AYP, PZ, NTT and JHS performed the analyses and visualisation. SM developed the KAS‐seq protocol. SN wrote the original and revised draft of the manuscript. All authors contributed to the review and editing of the manuscript.

## Supporting information


**Fig. S1.** InfoGain and Logmc‐derived eRNA overlaps for breast cancer subtypes, Logmc metrics, and UMAPs.
**Fig. S2**. Heatmaps showing eRNA expression in log2‐transformed mean‐recentred values from TCGA on Logmc and InfoGain‐derived eRNA regions.
**Fig. S3**. Infogain‐derived mRNA regions and ProxCReAm heatmaps, UMAPs, PCAs and visualisation of enriched terms.
**Fig. S4**. Visualisation of InfoGain and Logmc‐derived eRNAs compared to other datasets, (including good outcome, poor outcome primary tumours and metastatic samples from ER+ patients) as well as HiC datasets.
**Fig. S5**. Visualisation of overlaps between eRNAs and ATAC‐seq peaks, as well transcription factor motif enrichment.
**Fig. S6**. Heatmap of InfoGain‐derived survival‐specific eRNAs and associated metrics, as well as heatmap of prognostic eRNAs and visualisation of genomic distribution of eRNA loci.

## Data Availability

KAS‐seq data sets are available under the Gene Expression Omnibus accession number GSE289977.
