# Supplementary material for: Subtype‐specific enhancer RNAs define transcriptional regulators and prognosis in breast cancers
Source: Mol Oncol. 2026 Mar 2;20(7):1835–56. doi: 10.1002/1878-0261.70214 (PMC13352962; doi:10.1002/1878-0261.70214)
Supplement: Supplementary file 1 — Fig. S1. InfoGain and Logmc‐derived eRNA overlaps for breast cancer subtypes, Logmc metrics, and UMAPs. Fig. S2. Heatmaps showing eRNA expression in log2‐transformed mean‐recentred values from TCGA on Logmc and InfoGain‐derived eRNA regions. Fig. S3. Infogain‐derived mRNA regions and ProxCReAm heatmaps, UMAPs, PCAs and visualisation of enriched terms. Fig. S4. Visualisation of InfoGain and Logmc‐derived eRNAs compared to other datasets, (including good outcome, poor outcome primary tumours and metastatic samples from ER+ patients) as well as HiC datasets. Fig. S5. Visualisation of overlaps between eRNAs and ATAC‐seq peaks, as well transcription factor motif enrichment. Fig. S6. Heatmap of InfoGain‐derived survival‐specific eRNAs and associated metrics, as well as heatmap of prognostic eRNAs and visualisation of genomic distribution of eRNA loci. [file MOL2-20-1835-s001.pdf]

Supplementary Figures

Figure S1

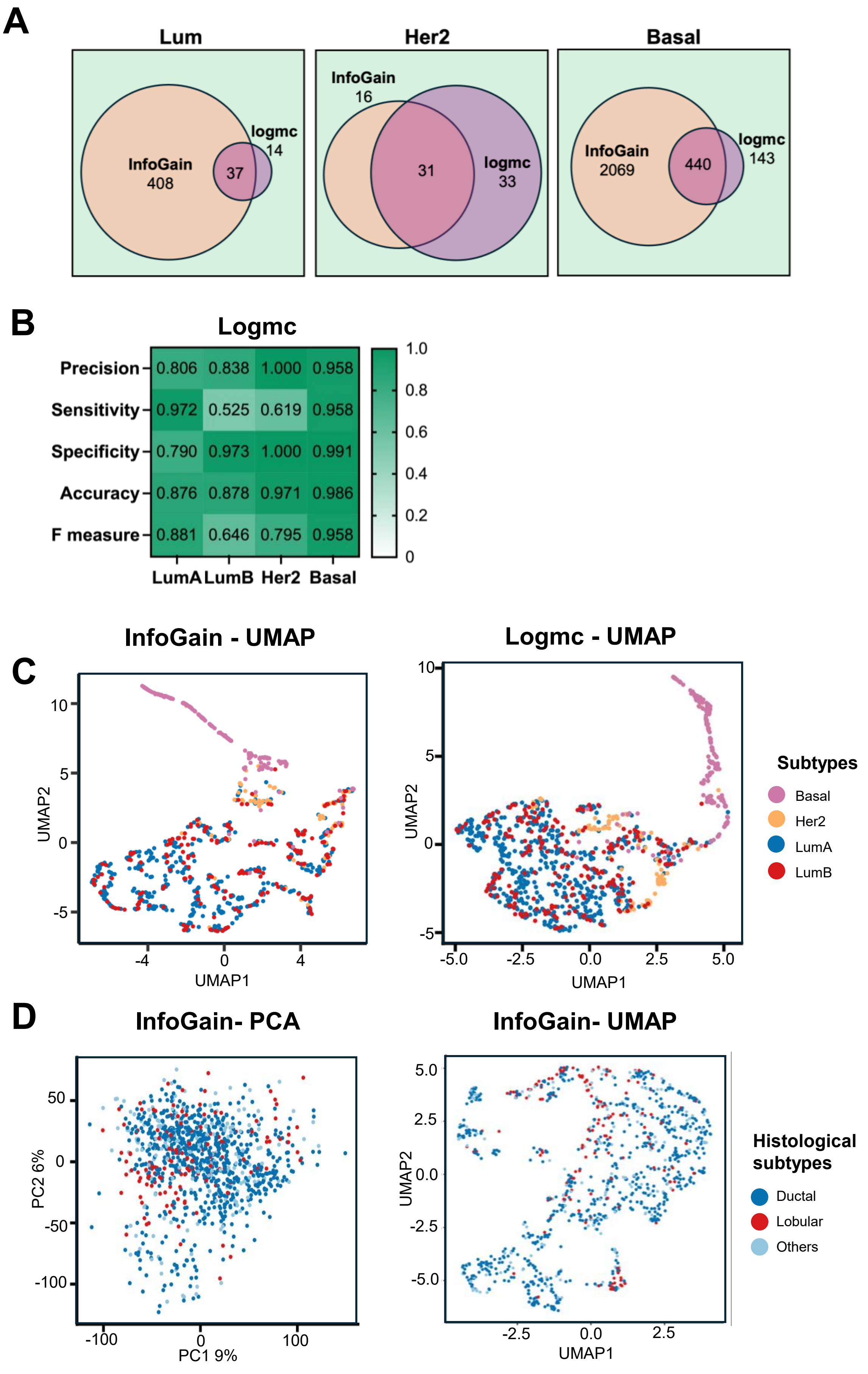

Figure S1: InfoGain and Logmc-derived eRNA overlaps for breast cancer subtypes, Logmc metrics, and UMAPs. **(A)** Venn diagram showing the overlap of all InfoGain and Logmc-derived eRNA regions from each subtype. Luminal A eRNAs are used for the overlap, as InfoGain could not identify any luminal B specific eRNAs. **(B)** Heatmap showing the statistics measures such as precision, sensitivity, specificity, accuracy and F-measure for log2-transformed mean centring (Logmc) measurements classifying luminal subtypes separately (luminal A and luminal B, as well as Her2 and basal). **(C)** UMAP analysis showing the efficiency of InfoGain- (top 4 Principal Components (PCs)) and Logmc- (top 2 PCs) derived eRNAs in classifying the clusters of patients from each subtype. **(D)** PCA and UMAP analysis showing the top 2 PCs (PCA) and top 4 PCs (UMAP) in classifying the clusters of patients from each histological subtype- invasive ductal, invasive lobular and others which includes mixed histology (NOS), mucinous, medullary and metaplastic carcinomas. As the classification did not yield enough ductal or lobular-specific eRNAs (n=1), PC components were made based on all the eRNAs filtered out of outliers, to show their indistinguishable nature.

Figure S2

A

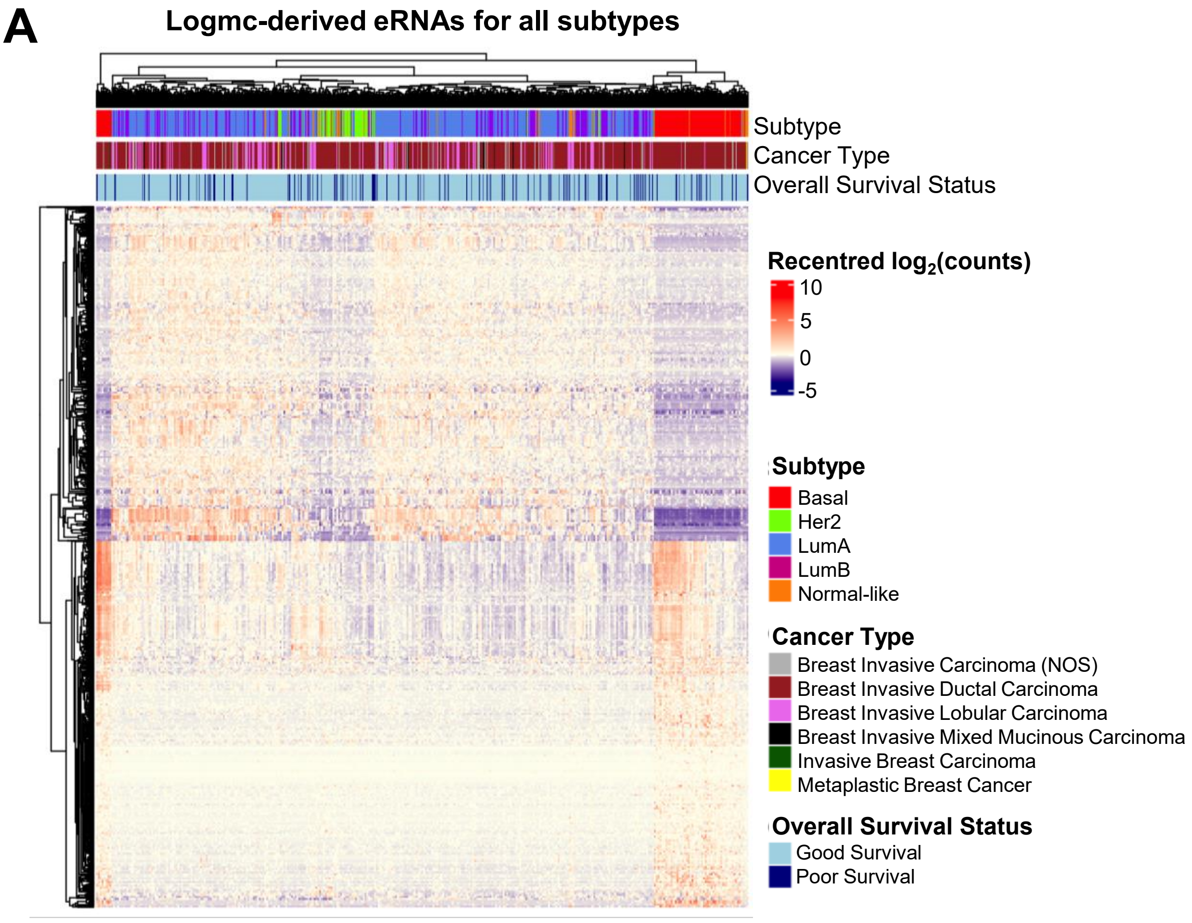

B

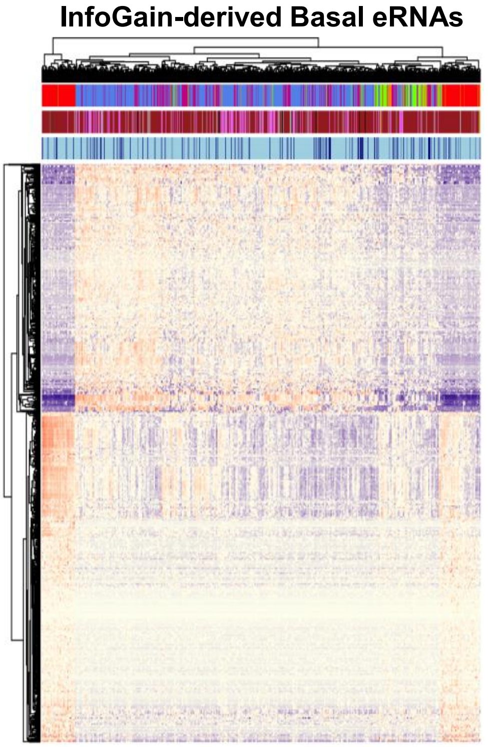

C

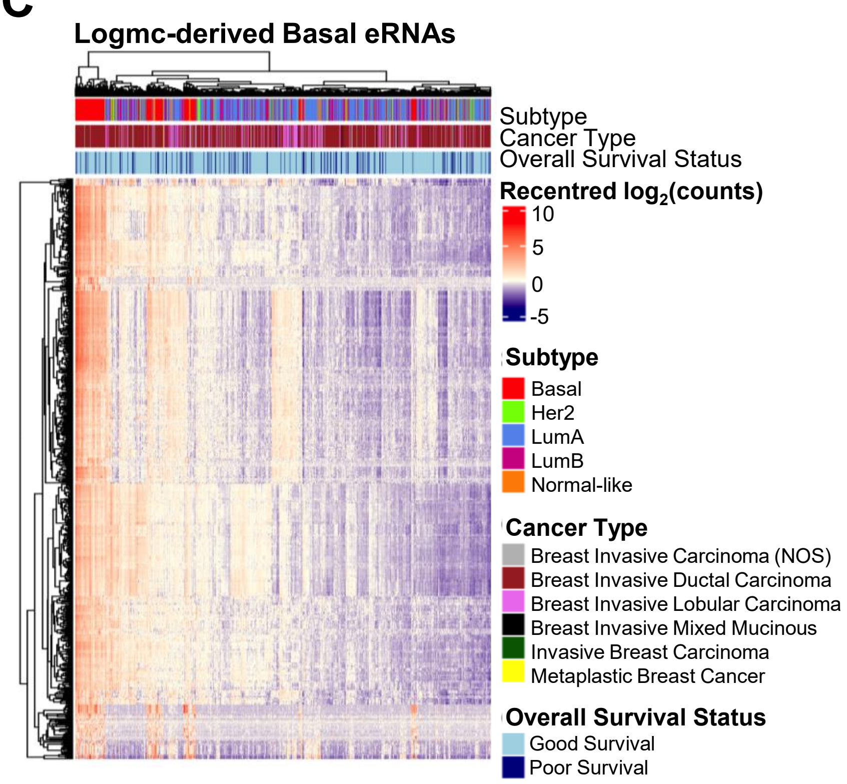

Figure S2 cont.

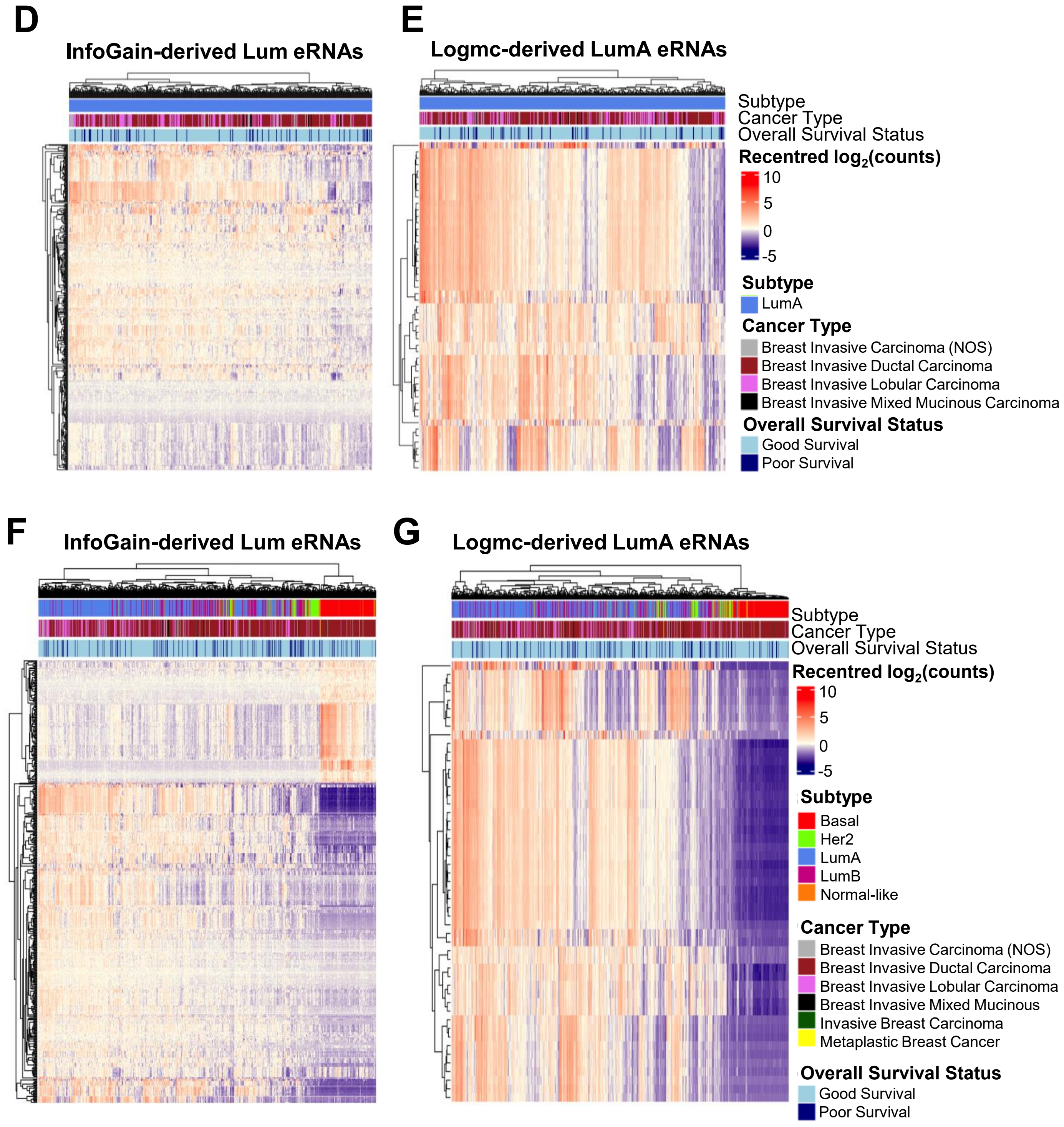

Figure S2 cont.

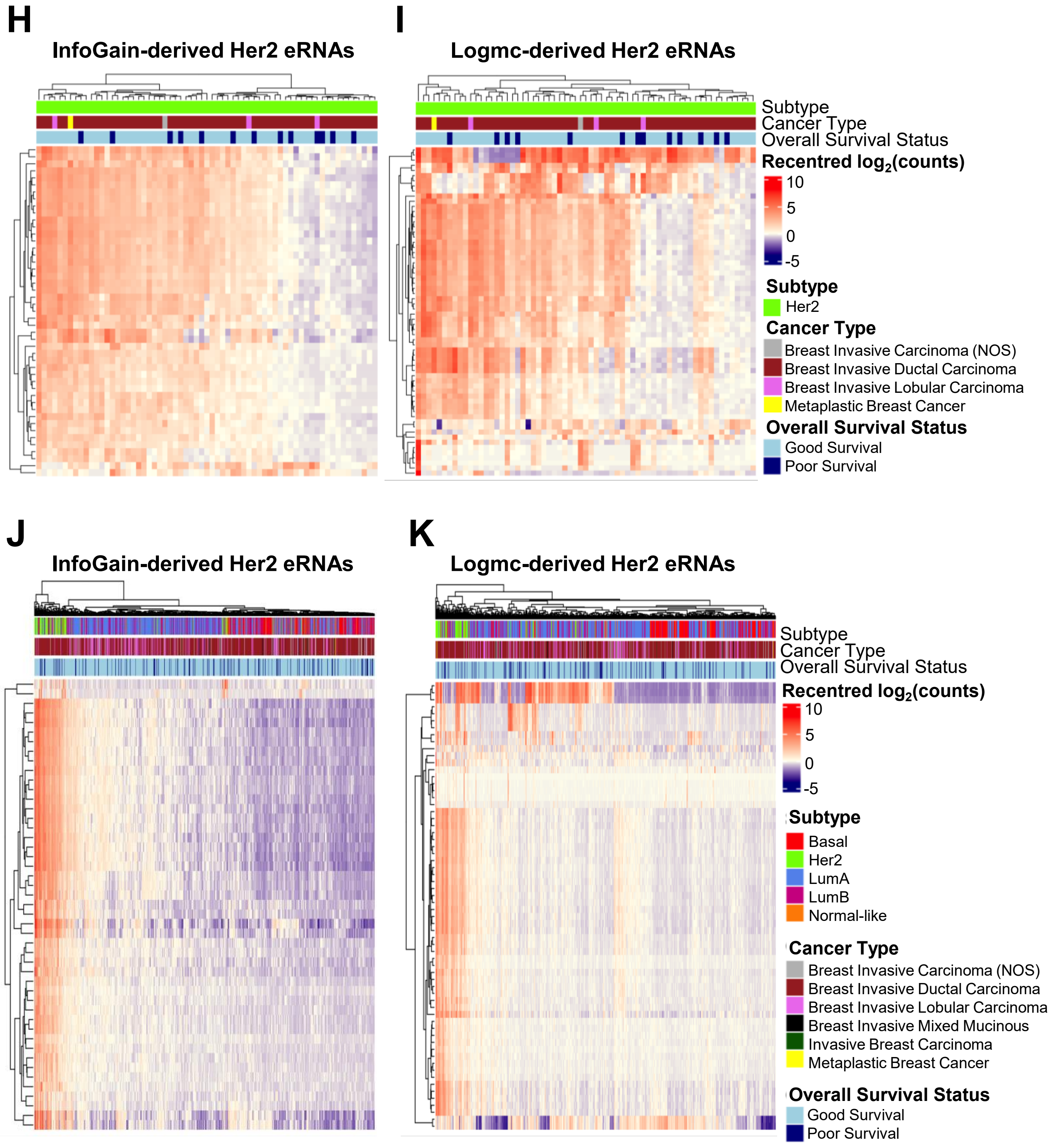

Figure S2 cont.

L

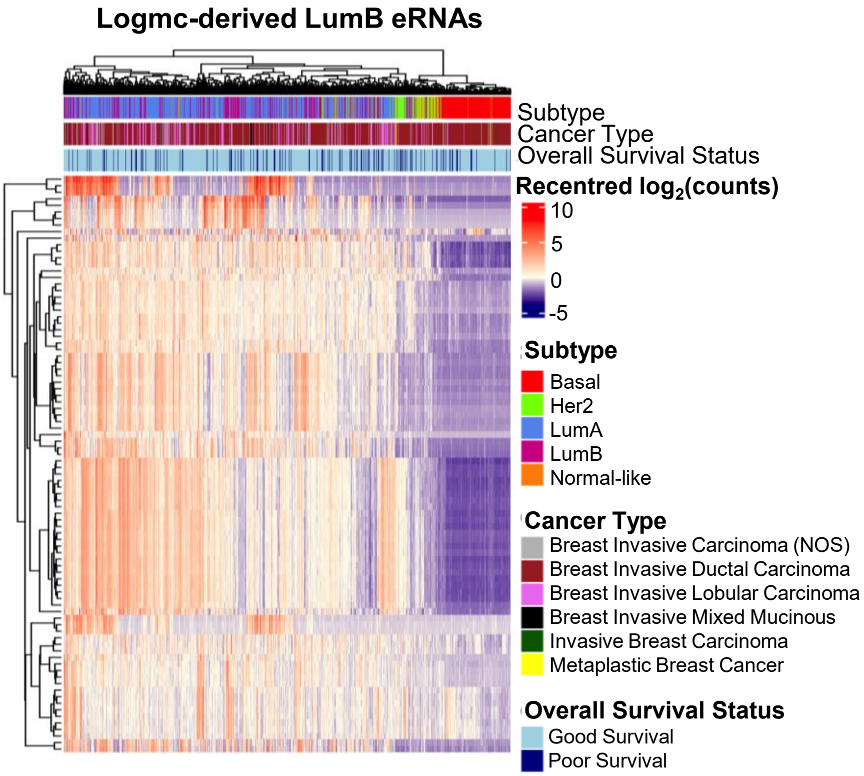

Figure S2: Heatmaps showing eRNA expression in log2-transformed mean-recentred values from TCGA on Logmc and InfoGain-derived eRNA regions. **(A)** Heatmap showing the eRNA expression in log2-transformed mean-recentred values of all breast cancer patient samples from TCGA on Logmc-derived eRNA regions from all subtypes. Annotation of each patient with molecular subtypes, histological origin-based cancer type (cancer type) and overall survival status is shown. **(B-K)** Heatmap showing eRNA expression in log2-transformed mean-recentred values of Basal (B-C), luminal A (**D-G**) and Her2 (**H-K**) patient samples, on InfoGain **(B, D, F, H, J)** and Logmc **(C, E, G, I, K)**- derived eRNA regions in respective subtype patients (**D, E, H, I**) or all patients (**B, C, F, G, J, K**). Annotation of each patient with molecular subtypes, histological cancer type and overall survival status is shown. **(L)** Heatmap showing eRNA expression in log2-transformed mean-recentred values of all breast cancer patient samples from TCGA on LumB-specific Logmc-derived eRNA.

Figure S3

A

InfoGain-derived mRNAs for all subtypes

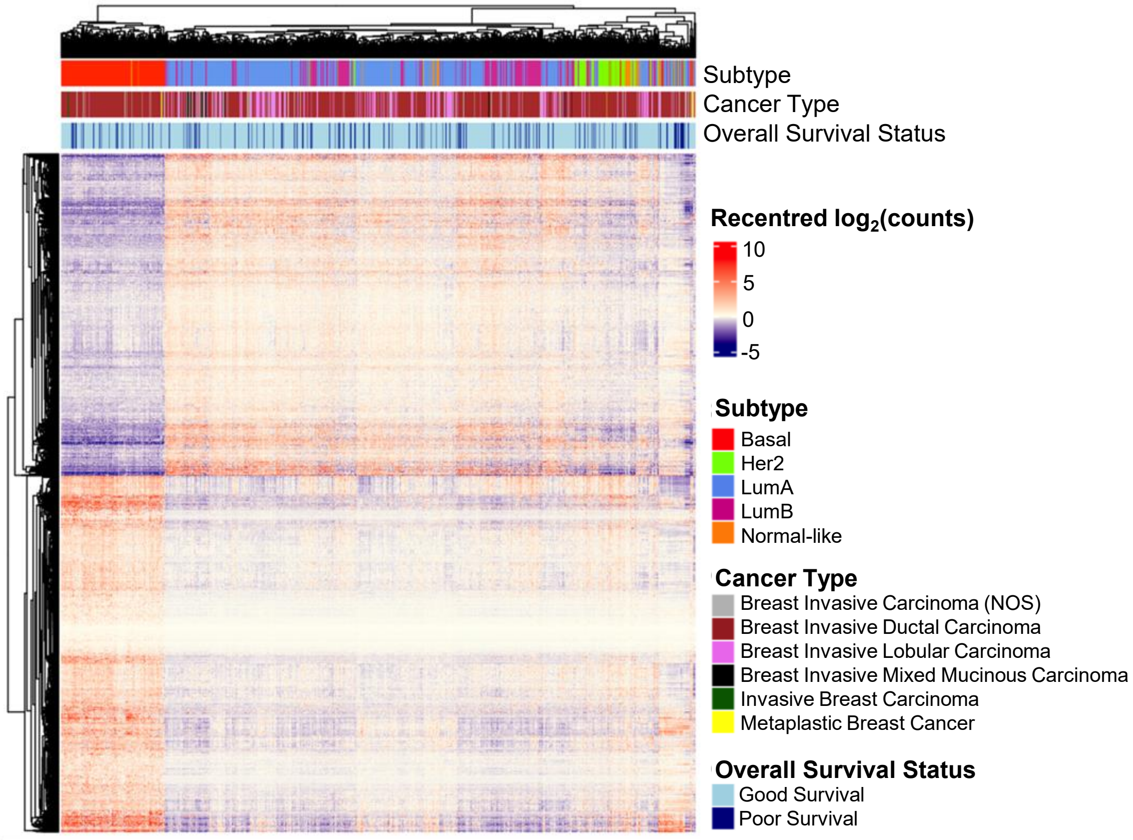

B

ProxCREAM eRNAs for all subtypes

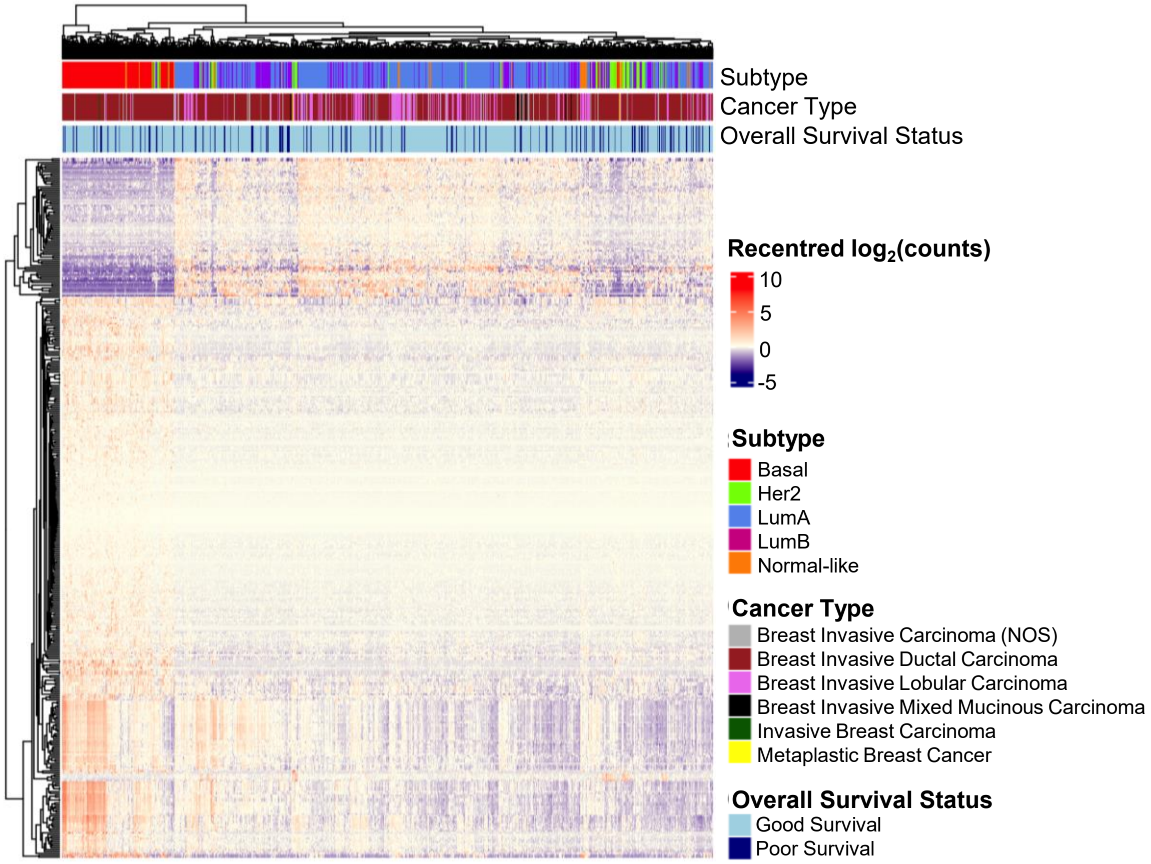

Figure S3 cont

C

Subtype InfoGain mRNAs

Measure	Basal	Her2	Lum A	Lum B
InfoGain	2181	13	1225	9
No. of patients	194	87	568	220

D

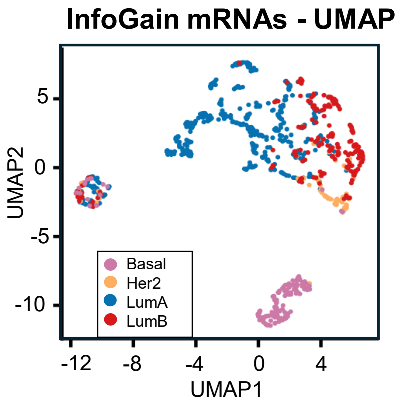

E

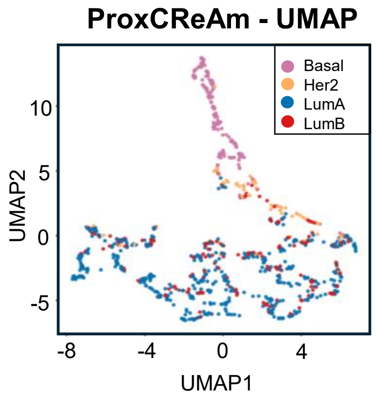

F

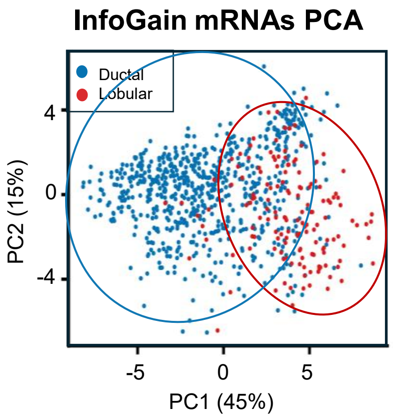

G

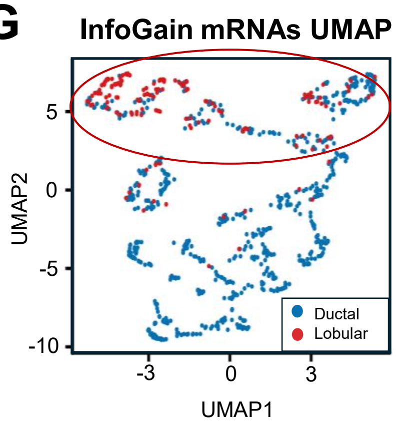

H

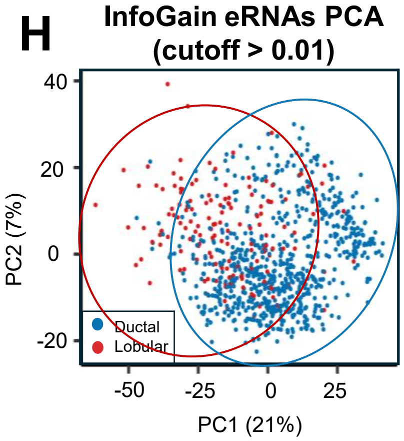

I

Ductal/lobular ProxCReAm association

Subtype-specific eRNAs

Distance in bps	Subtype-specific mRNAs
150	1
300	1
500	1
700	2
1000	2
5000	2
10000	3
50000	3
100000	10
200000	19
500000	30
1000000	33
1500000	34
2000000	35

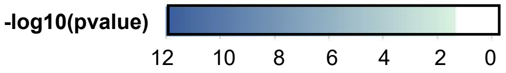

J

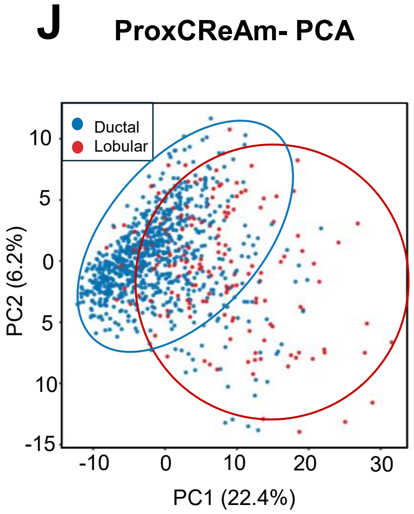

K

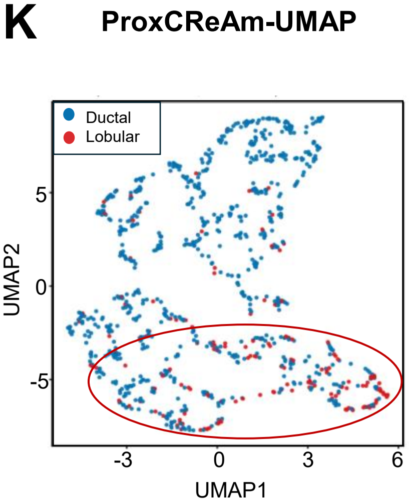

Figure S3 cont

L

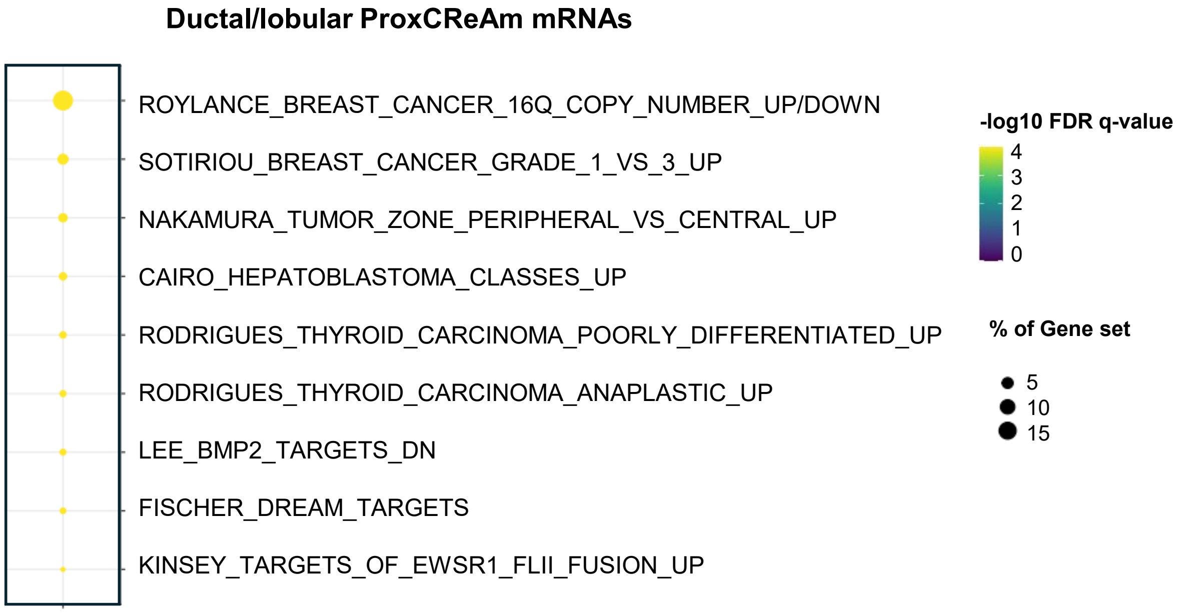

Figure S3: Infogain-derived mRNA regions and ProxCReAm heatmaps, UMAPs, PCAs and visualisation of enriched terms. **(A)** Heatmap showing the mRNA expression in log2-transformed mean-recentred values of all breast cancer patient samples from TCGA on Infogain-derived mRNA regions from all subtypes. Annotation of each patient with molecular subtypes, histological origin-based cancer type (cancer type) and overall survival status is shown. **(B)** Heatmap showing proximally expressed and subtype-specific mRNA-associated eRNA (ProxCReAm) expression in log2-transformed mean-recentred values of all breast cancer patient samples from TCGA paired with InfoGain measure from all subtypes. Annotation of each patient with molecular subtypes, histological cancer type and overall survival status is shown. **(C)** Number of mRNA regions classified per subtype with InfoGain measurement is shown in a table. **(D)** UMAP analysis showing the efficiency of InfoGain- (top 4 Principal Components (PCs)) derived mRNAs in classifying the clusters of patients from each subtype. **(E)** UMAP analysis showing the efficiency of InfoGain- (top 4 Principal Components (PCs)) derived ProxCReAm eRNAs in classifying the clusters of patients from each subtype. **(F)** PCA analysis showing the efficiency of InfoGain-derived mRNAs, using the top 2 principal components, in classifying the clusters of patients from each histological subtype- invasive ductal and invasive lobular. Other subtypes with mixed histology (NOS), mucinous, medullary and metaplastic carcinomas were excluded. Blue oval shows ductal sample distribution and red oval shows lobular distribution. **(G)** UMAP analysis showing the efficiency of InfoGain-derived mRNAs, using the top 4 principal components, in classifying the clusters of patients from each histological subtype- invasive ductal and invasive lobular. Red oval shows lobular distribution. **(H)** PCA analysis showing the efficiency of InfoGain-derived eRNAs with cutoff 0.01 (n=7440), using the top 2 principal components, in classifying the clusters of patients from each histological subtype- invasive ductal and invasive lobular. InfoGain measure above 0.01 was taken forward for the analyses resulting in 7440 regions. Blue oval shows ductal sample distribution and red oval shows lobular distribution. **(I)** Heatmap showing the details of PEGS results to identify the ProxCReAm eRNAs, where InfoGain-derived eRNAs with cutoff 0.01 are proximally associated with mRNAs, to separate invasive ductal and invasive lobular histological subtypes. The number represents the ProxCReAm mRNAs with different distances (from 150 bp – 2 MB) and the colour represents hypergeometric –log10p-value. **(J)** PCA analysis showing the efficiency of InfoGain-derived ProxCReAm eRNAs (n=223) with cutoff 0.01, using the top 2 principal components, in classifying the clusters of patients from each histological subtype- invasive ductal and invasive lobular. Blue oval shows ductal sample distribution and red oval shows lobular distribution. **(K)** UMAP analysis showing the efficiency of InfoGain-derived ProxCReAm eRNAs (n=223) with cutoff 0.01, using the top 4 principal components, in classifying the clusters of patients from each histological subtype- invasive ductal and invasive lobular. Red oval shows lobular distribution. **(L)** Dotplot showing gene pathway analyses performed on InfoGain-derived ProxCReAm mRNAs (n=33) of each histological subtype- invasive ductal and invasive lobular using MSigDB database (CGP, C5, C6 gene sets). Colour keys represent -log10 FDR q-value and percentage of gene set (percentage of the ratio of overlapped genes by total number of genes in a pathway gene set). Only significant pathways are shown.

Figure S4

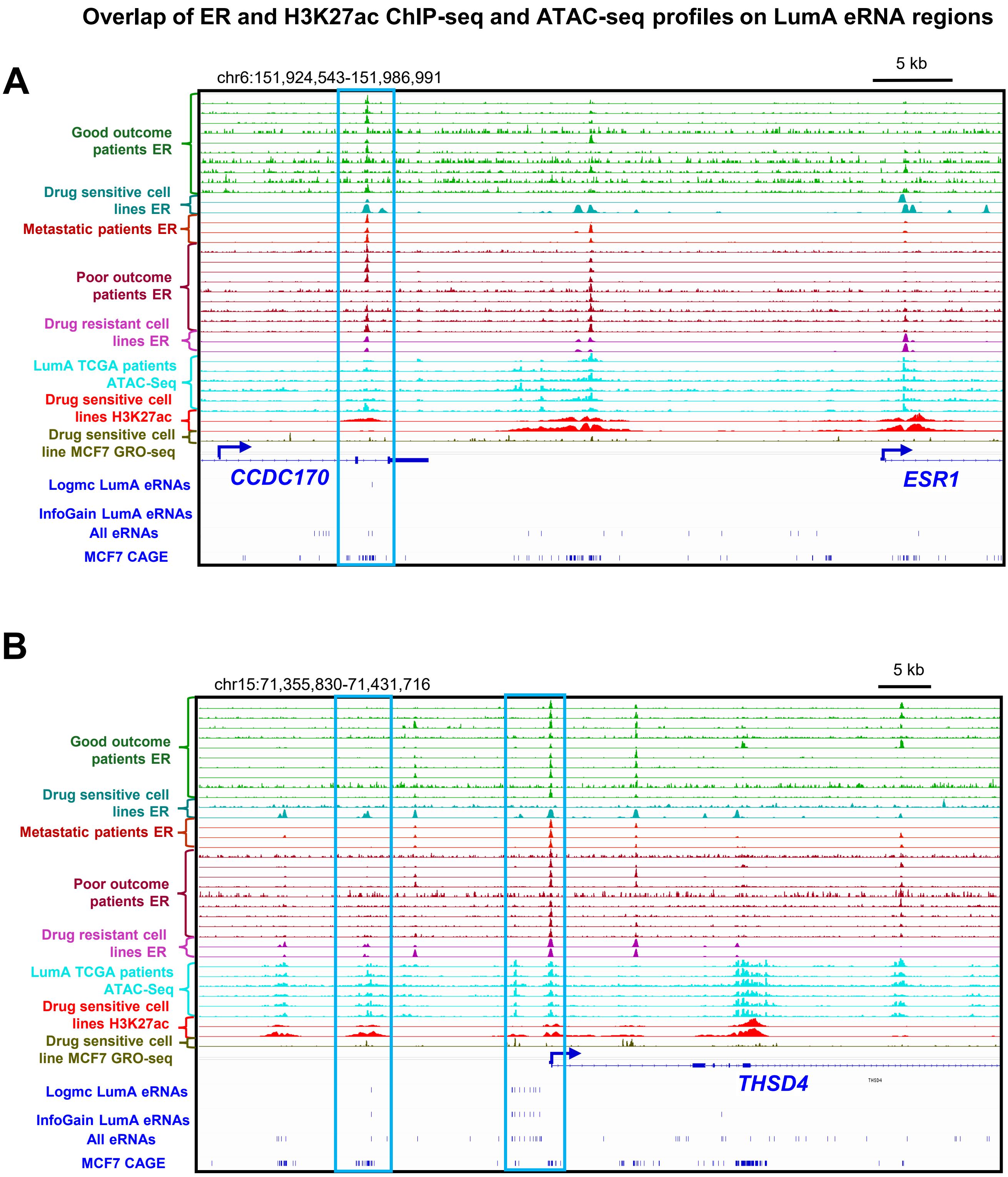
Overlap of ER and H3K27ac ChIP-seq and ATAC-seq profiles on LumA eRNA regions
A
chr6:151,924,543-151,986,991
5 kb
Good outcome patients ER
Drug sensitive cell lines ER
Metastatic patients ER
Poor outcome patients ER
Drug resistant cell lines ER
LumA TCGA patients ATAC-Seq
Drug sensitive cell lines H3K27ac
Drug sensitive cell line MCF7 GRO-seq
CCDC170
ESR1
Logmc LumA eRNAs
InfoGain LumA eRNAs
All eRNAs
MCF7 CAGE
B
chr15:71,355,830-71,431,716
5 kb
Good outcome patients ER
Drug sensitive cell lines ER
Metastatic patients ER
Poor outcome patients ER
Drug resistant cell lines ER
LumA TCGA patients ATAC-Seq
Drug sensitive cell lines H3K27ac
Drug sensitive cell line MCF7 GRO-seq
THSD4
Logmc LumA eRNAs
InfoGain LumA eRNAs
All eRNAs
MCF7 CAGE

Figure S4 cont

Overlap of ER and H3K27ac ChIP-seq and ATAC-seq profiles on LumA eRNA regions

C

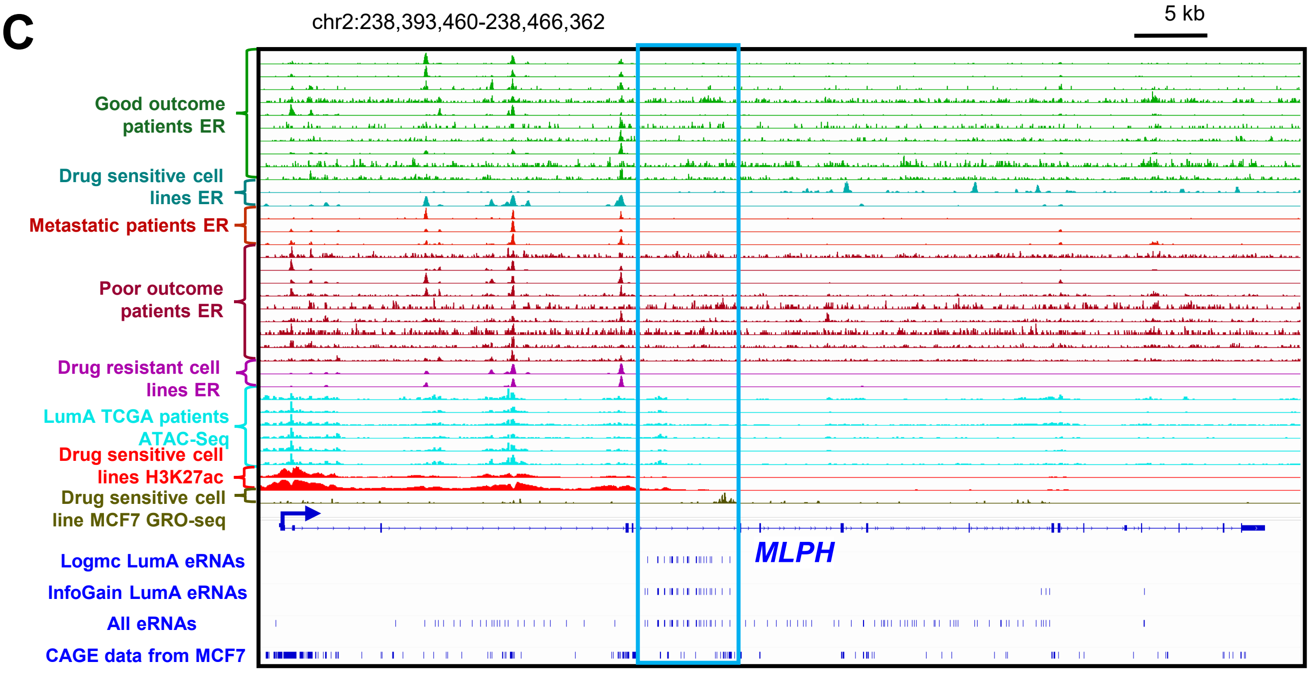

D

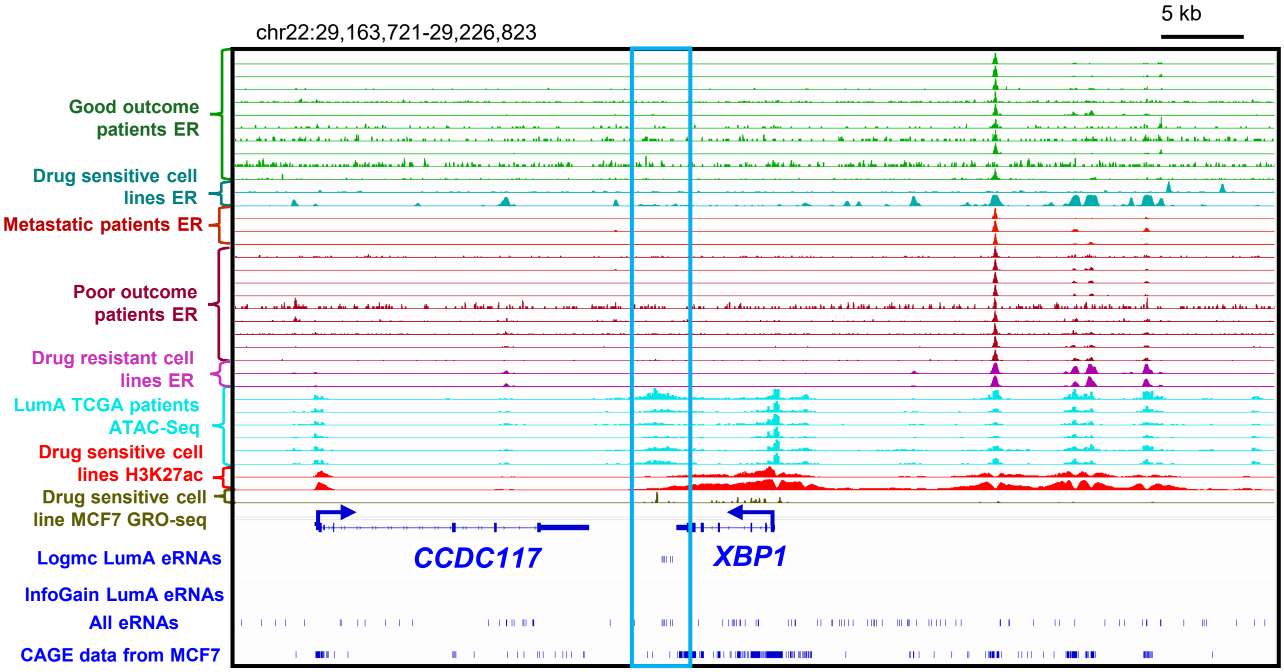

Figure S4 cont

E

HCC1954 (Her2+ cell line)

hg38: chr17:39,208,376-40,208,376, 5 kb resolution

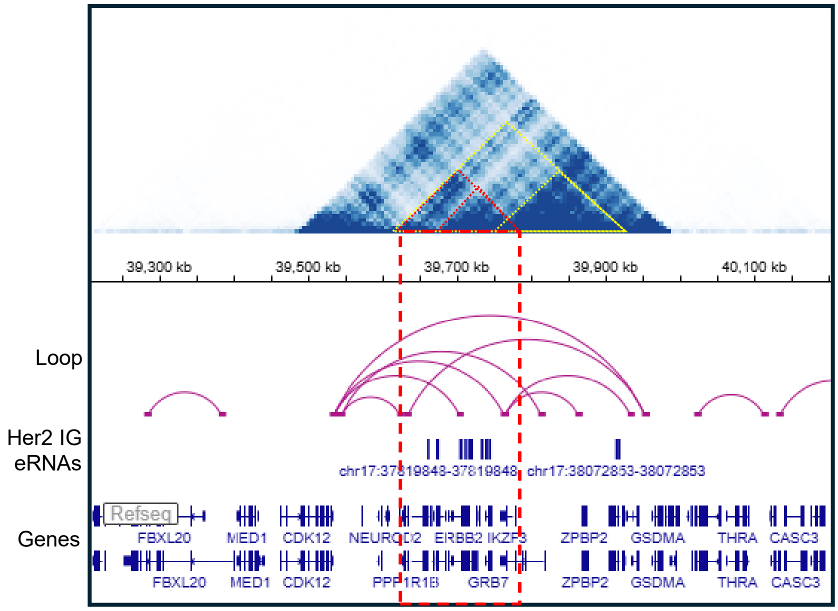

F

BT-474 (ER+ PR+ Her2+ cell line)

hg38: chr17:39,208,376-40,208,376, 5 kb resolution

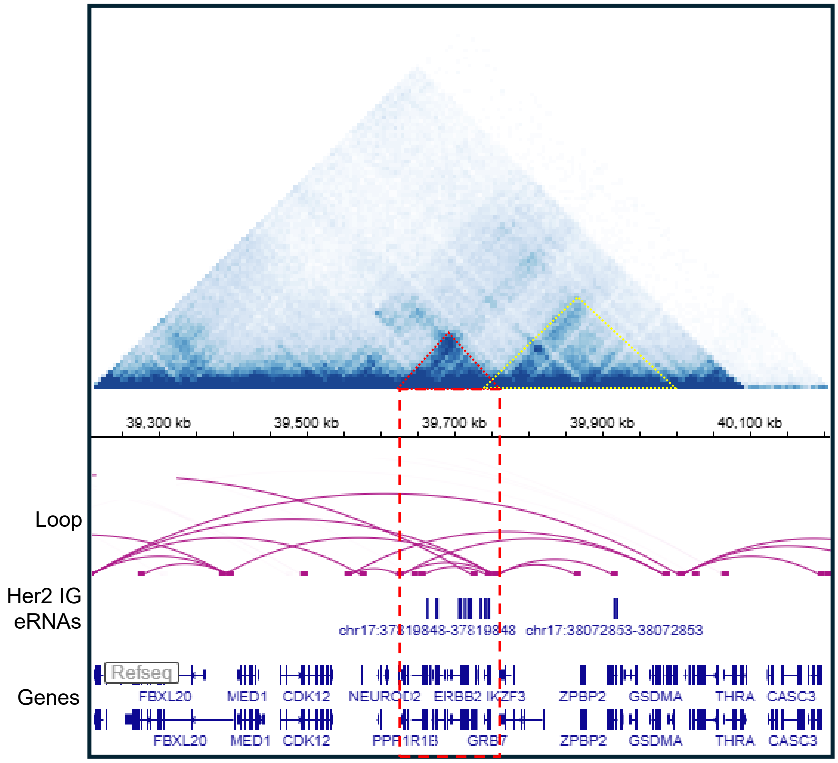

Figure S4: Visualisation of InfoGain and Logmc-derived eRNAs compared to other datasets, (including good outcome, poor outcome primary tumours and metastatic samples from ER+ patients) as well as HiC datasets. **(A-D)** Multiple gene view close to *ESR1* (ER) **(A)**, THSD4 **(B)**, MLPH **(C)** and XBP1 **(D)** genes showing the occupancy of ER (blue rectangles) in: good outcome (green) and poor outcome primary tumour (brown) and metastatic samples (dark brown) from ER+ patients; cell lines representing drug sensitive (MCF7, ZR-75-1, both in teal); global run-on sequencing (GRO-seq) signals from MCF7 cells representing drug sensitive luminal A cell line (olive green); tamoxifen-resistant (BT474, MCF7 Tamoxifen-resistant TamR) cell lines (purple); ATAC-seq regions from luminal A ER+ patients (blue); and H3K27ac ChIP-seq signals from drug sensitive luminal A cell lines MCF7 and ZR-75-1 (red). These were compared to eRNA regions derived from Logmc and InfoGain-specific to luminal A subtype as well as all eRNA regions, as defined by Chen *et al.,* 2018 (300K eRNA loci) and cap analysis of gene expression (CAGE) data from MCF7 cells. **(E-F)** Visualisation of the HiC datasets from two different Her2-enriched cell lines on the Her2-specific InfoGain-derived eRNA regions on the locus chr17:39,208,376-40,208,376 at 5 kb resolution. HCC1954 **(E)** is a Her2+ ER/PR - breast cancer cell line and BT474 **(F)** is ER/PR/Her2 + breast cancer cell line. HiC-derived topologically associated domains (TADs) are shown as heatmaps and loops re shown in pink. The TAD directly associated with Her2 eRNAs and *ERBB2* gene is highlighted in red and other surrounding TADs in yellow.

Figure S5

A

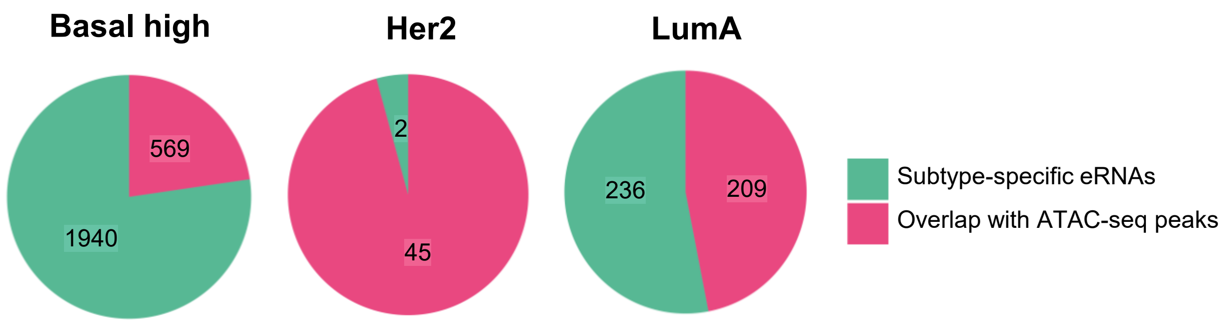

B

Measurement	Basal high	Her2	LumA
InfoGain	2509	47	445
eRNA regions overlapping with ATAC-seq peaks	569	45	209
Unique eRNA regions overlapping with ATAC-seq peaks	253	4	85

C

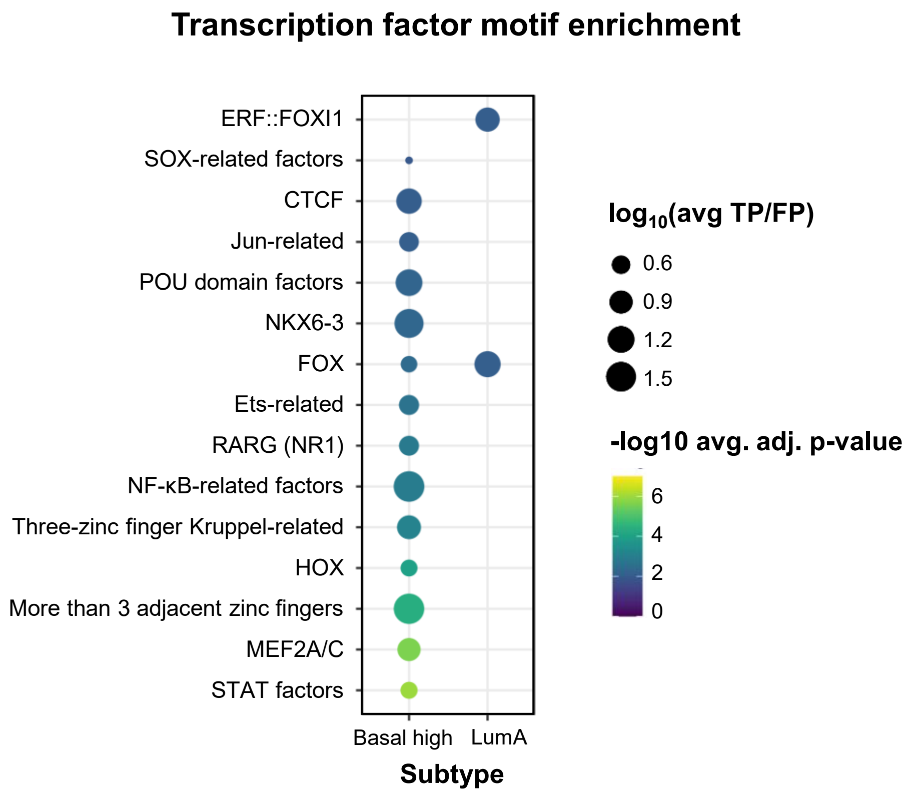

Figure S5: Visualisation of overlaps between eRNAs and ATAC-seq peaks, as well transcription factor motif enrichment. **(A)** Pie charts showing the overlap (pink) of TCGA ATAC-seq peaks from each breast cancer subtype (basal, Her2 and Luminal A) with the subtype-specific eRNA regions. Non-overlapped regions are shown in green. **(B)** Number of eRNA regions classified per subtype with InfoGain measurement, their overlap with TCGA ATAC-seq peaks from each subtype and the unique overlapping peaks. **(C)** Dotplot showing motif enrichment analyses performed on overlapped ATAC-seq peaks with InfoGain-derived eRNA regions (1000 bp flanks) of basal subtype with high expression (Basal high, n=63) and luminal A-specific (n=19) regions. Colour keys represent log10 average of the ratio of true positives over false positive motifs and -log10 of the average of adjusted p-value per motif family from AME tool from the MEME suite using JASPAR 2022 Core vertebrates non-redundant v2 database. Only significant pathways are shown. Her2-eRNA and ATAC-seq regions did not overlap on any sites. TF motifs were sorted and visualised using the average -log10 adjusted p-value per subtype.

Figure S6

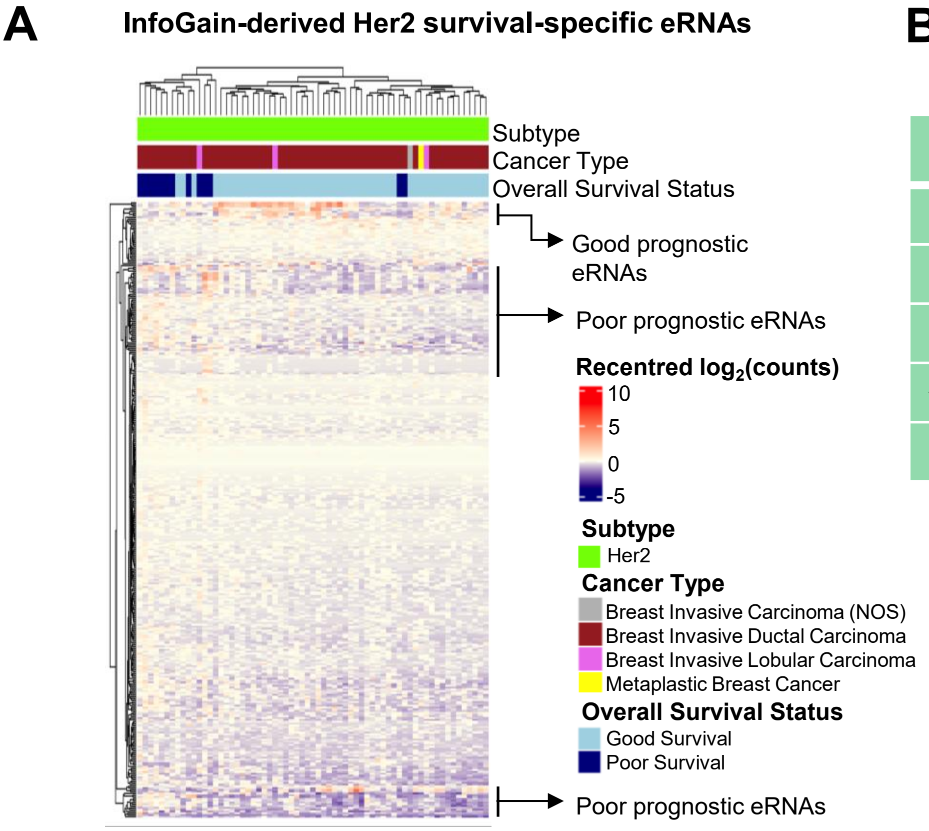

B

Metrics	Good outcome	Poor outcome
Precision	0.941	0.833
Sensitivity	0.941	0.833
Specificity	0.857	0.944
Accuracy	0.957	0.957
F-measure	0.941	0.833

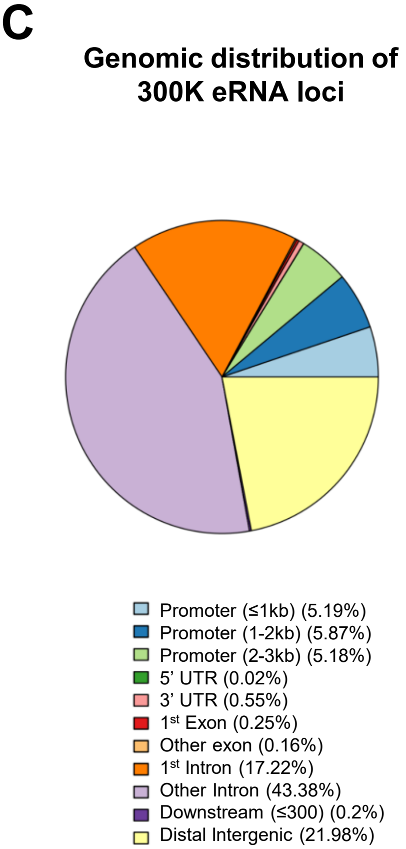

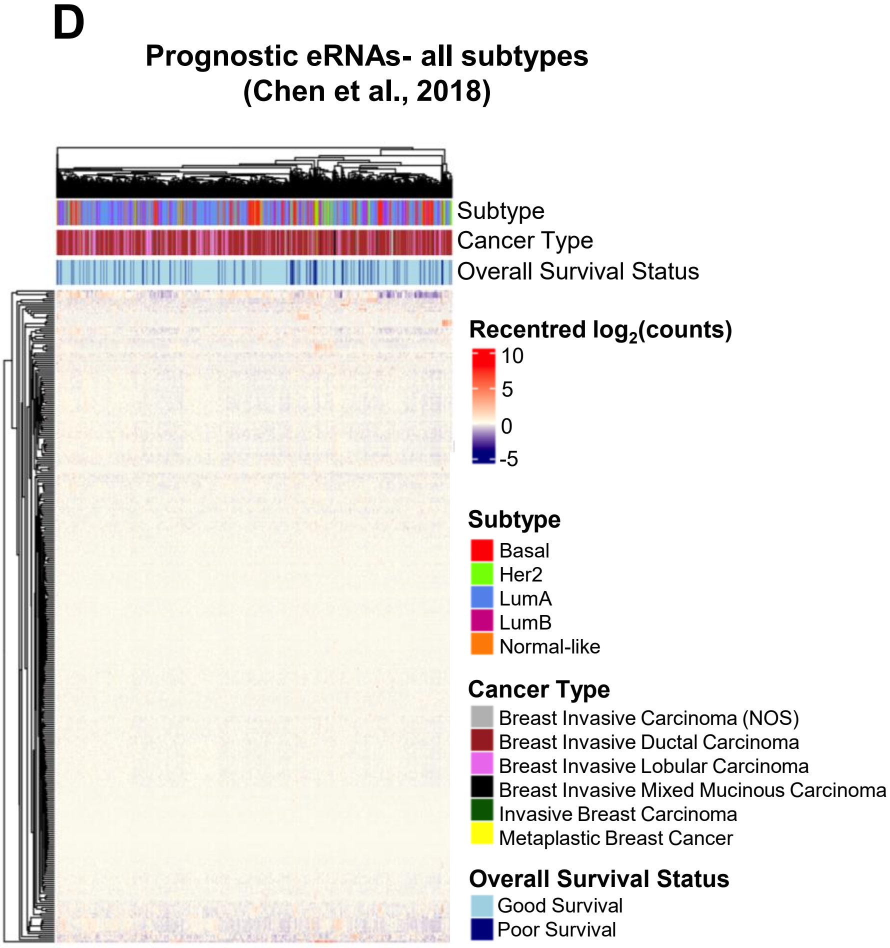

Figure S6: Heatmap of InfoGain-derived survival-specific eRNAs and associated metrics, as well as heatmap of prognostic eRNAs (Chen *et al.,* 2018) and visualisation of genomic distribution of eRNA loci. **(A)** Heatmap showing eRNA expression in log2-transformed mean-recentred values on InfoGain-derived Her2 and survival-specific eRNA regions. Annotation of each patient with molecular subtypes, cancer type and overall survival status is shown. eRNA loci associated with good and poor survival are marked with arrows. (**B**) Table showing the statistics measures such as precision, sensitivity, specificity, accuracy and F-measure for information gain (InfoGain) measurements classifying Her2 subtype based on survival (good and poor outcome patients). **(C)** Pie chart showing the genomic distribution of eRNA loci showing that the majority of eRNAs are located in intronic or intergenic regions. **(D)** Heatmap showing eRNA expression in log2-transformed mean-recentred values in rpkm on prognostic eRNA loci (n= 326) identified by Chen *et al*., 2018. Annotation of each patient with molecular subtypes, cancer type and overall survival status is shown.
